# Incidence and Risk Factors of Cancer in the Anal Transitional Zone and Ileal Pouch following Surgery for Ulcerative Colitis and Familial Adenomatous Polyposis

**DOI:** 10.3390/cancers14030530

**Published:** 2022-01-21

**Authors:** Guillaume Le Cosquer, Etienne Buscail, Cyrielle Gilletta, Céline Deraison, Jean-Pierre Duffas, Barbara Bournet, Géraud Tuyeras, Nathalie Vergnolle, Louis Buscail

**Affiliations:** 1 Department of Gastroenterology and Pancreatology, CHU Toulouse-Rangueil (University Hospital Centre) and Toulouse University, UPS, 31059 Toulouse, France; lecosquer.g@chu-toulouse.fr (G.L.C.); gilletta.c@chu-toulouse.fr (C.G.); bournet.b@chu-toulouse.fr (B.B.); 2 Department of Surgery, CHU Toulouse-Rangueil and Toulouse University, UPS, 31059 Toulouse, France; ebuscail@me.com (E.B.); duffas.jp@chu-toulouse.fr (J.-P.D.); tuyeras.g@chu-toulouse.fr (G.T.); 3 IRSD, Toulouse University, INSERM 1022, INRAe, ENVT, UPS, 31300 Toulouse, France; celine.deraison@inserm.fr (C.D.); nathalie.vergnolle@inserm.fr (N.V.); 4 Centre for Clinical Investigation in Biotherapy, CHU Toulouse-Rangueil and INSERM U1436, 31059 Toulouse, France

**Keywords:** anal transitional zone cancer, ileal pouch, ulcerative colitis, familial adenomatous polyposis, high grade dysplasia

## Abstract

**Simple Summary:**

Proctocolectomy with ileal pouch-anal anastomosis is the intervention of choice for ulcerative colitis and familial adenomatous polyposis requiring surgery. However high-grade dysplasia and cancer in the anal transitional zone and ileal pouch after 20 years is estimated to be 2 to 4.5% and 3 to 10% in ulcerative colitis and familial polyposis, respectively. The risk factors for ulcerative colitis are the presence of pre-operative dysplasia or cancer, disease duration > 10 years and severe villous atrophy. For familial polyposis, the risk factors are the number of pre-operative polyps > 1000, surgery with stapled anastomosis and the duration of follow-up. Even if anal transitional zone and ileal pouch cancers seldom occur following proctectomy for ulcerative colitis and familial adenomatous polyposis, the high mortality rate associated with this complication warrants close endoscopic monitoring, mainly every year with pouchoscopy including chromoendoscopy.

**Abstract:**

Proctocolectomy with ileal pouch-anal anastomosis is the intervention of choice for ulcerative colitis and familial adenomatous polyposis requiring surgery. One of the long-term complications is pouch cancer, having a poor prognosis. The risk of high-grade dysplasia and cancer in the anal transitional zone and ileal pouch after 20 years is estimated to be 2 to 4.5% and 3 to 10% in ulcerative colitis and familial polyposis, respectively. The risk factors for ulcerative colitis are the presence of pre-operative dysplasia or cancer, disease duration > 10 years and severe villous atrophy. For familial polyposis, the risk factors are the number of pre-operative polyps > 1000, surgery with stapled anastomosis and the duration of follow-up. In the case of ulcerative colitis, a pouchoscopy should be performed annually if one of the following is present: dysplasia and cancer at surgery, primary sclerosing cholangitis, villous atrophy and active pouchitis (every 5 years without any of these factors). In the case of familial polyposis, endoscopy is recommended every year including chromoendoscopy. Even if anal transitional zone and ileal pouch cancers seldom occur following proctectomy for ulcerative colitis and familial adenomatous polyposis, the high mortality rate associated with this complication warrants endoscopic monitoring.

## 1. Introduction

Restorative proctocolectomy (RPC) with ileal pouch-anal anastomosis (IPAA) is the intervention of choice for familial adenomatous polyposis (FAP) and ulcerative colitis (UC) requiring surgery [1]. UC is characterised by chronic mucosal inflammation of the rectum and colon [2]. The two peaks for UC diagnosis are between 15 and 30 years of age with a second smaller peak after 60 years of age [3]. The colectomy rate 10 years post-diagnosis has recently been estimated to be 15.6% [4]. The main indications for surgery are endoscopically unresectable dysplasia (or cancer), chronic colitis refractory to medical management, complicated (uncontrolled haemorrhage, perforation) and refractory acute severe colitis [5]. FAP is an inherited autosomal dominant disease associated with predisposition to colorectal cancer, with a prevalence of 1 in 10,000 inhabitants [6]. This syndrome is caused by a germline mutation in the adenomatous polyposis coli gene (located on chromosome 5q21) [7]. The phenotypic presentation is characterised by the early onset of hundreds of adenomas leading to colorectal cancer by the age of 40 in almost 100% of cases. Prophylactic colectomy is the only treatment to reduce the colorectal cancer risk and should be offered to all patients between 15 and 25 years of age [8].

RPC consists of the removal of the rectum and the colon and the construction of an ileal pouch (reservoir formed with the last 40 cm of the ileum) followed by IPAA [9]. This surgery can be performed in one, two or three stages depending on the colectomy indication. In patients at high risk of complication (e.g., acute severe colitis), the first step is to remove the right, transverse and left colon (i.e., subtotal colectomy) with double-end ileostomy and sigmoidostomy [10]. Then, 3 to 6 months later, the rectum and sigmoid are removed and an IPAA is formed with a temporary diverting ileostomy to allow the anastomosis to heal. Finally, the stoma is reversed 6 to 8 weeks later. In the absence of local inflammation or factors indicative of complications, a two-stage procedure can be carried out. The first step is RPC with IPAA, and a temporary diverting ileostomy followed 6 to 8 weeks later by closure of the stoma. A single-stage technique is chosen by some surgeons for certain patients (young healthy patients, elective surgery) to avoid ileostomy.

The main complications reported can be divided into short-term (within 1 month after surgery) and long-term complications [11]. Short-term complications are acute bleeding, leakage, abscesses and small bowel obstruction. The long-term complications are incontinence, chronic pelvic sepsis, pouch stricture, decreased fertility, pouch failure (defined as the need to remove the pouch), pouch dysplasia and cancer [12]. Patients operated on for UC are also at risk of pouchitis (i.e., inflammation of the pouch) and development of Crohn’s disease in the pouch. The laparoscopic approach is to be preferred where possible, as it has been proven to lower the risk of postoperative complications and to preserve fertility [13,14].

**Figure 1:**
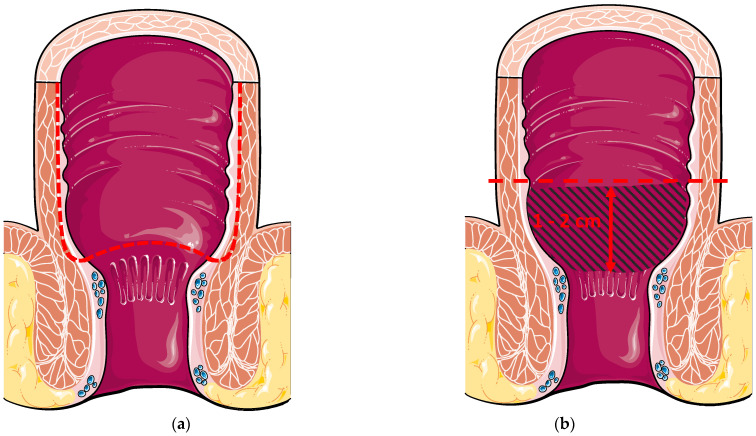
(**a**) Hand-sewn ileal pouch anal anastomosis with transanal mucosectomy. The dotted line represents the mucosectomy started above the dentate line; (**b**) Double-stapled ileal pouch anal anastomosis 1–2 cm above the dentate line. The blue hatched area represents the cuff rectal mucosa.

Ileoanal anastomosis is the critical part of the surgery. Two anastomotic techniques have been described, namely double-stapled or hand-sewn anastomosis (Figure 1) [15]. Manual (hand-sewn) anastomosis consists of creating an anastomosis between the pouch and the anal transitional zone (ATZ) just above the dentate line following endoanal mucosectomy [16,17]. The ATZ is defined as the area between the dentate line and the columnar epithelium, measuring 5–10 mm [18,19]. Histologically, this zone is characterised by the interchange between the squamous epithelium (from the anus) and the columnar epithelium (from the rectum) [20]. Conversely, a mechanical (double-stapled) anastomosis is created 1–2 cm above the ATZ, leaving a cuff of rectal mucosa [21]. The advantage of the manual technique is that any potential risk of diseased tissue (inflammation, dysplasia, cancer) is eradicated. The downside of the technique is an increased risk of leakage [22]. On the other hand, leaving the rectal cuff allows air-liquid/solid differentiation, thus lowering the risk of incontinence, which is a major issue for patients [23,24].

The epidemiology of these diseases implies that some patients undergo surgery at a young age. Balancing functional results and the risk of cancer in the ATZ and ileal pouch after surgery poses a real management challenge. Indeed, the rate of dysplasia of the ATZ after RPC for UC was estimated as 1.13% in a recent meta-analysis [25]. Tsunoda et al. described a higher rate of dysplasia in the case of FAP [26]. Cases of adenocarcinoma of the ATZ and pouch have been reported for both diseases [27,28]. Derikx et al. reported a cumulative incidence of 2% and 6% for dysplasia and cancer of the pouch per se, 10 and 20 years after RPC, respectively, [29]. The prognosis for pouch adenocarcinoma is poor. Wu et al. reported a global mortality rate of 42.9% 2 years after the diagnosis of pouch cancer [30]. Despite this known risk, few follow-up data are available for anastomosis. Those patients must be offered endoscopic follow-up to screen for dysplasia and cancer. The treatment proposed for cancer of the ATZ (or pouch) is pouch excision and terminal ileostomy. Endoscopic resection, notably by submucosal dissection, has emerged as an alternative curative treatment for endoscopically resectable lesions (dysplasia and cancer) [31,32].

The aim of this review is to report on the available data for dysplasia and cancer of the ATZ and pouch focusing on incidence, risk factors, the impact of surgery, in particular, and the initial pathology. This review will also outline the mechanisms leading to dysplasia, including the role of inflammation in the process of carcinogenesis and interactions between mucosa. Finally, we propose an algorithm for post-IPAA endoscopic monitoring.

## 2. Methods

A PubMed literature search for “pouch dysplasia”,” pouch cancer”, “pouch neoplasia”, “anal transitional zone dysplasia”, “anal transitional zone cancer”, “anal transitional zone neoplasia”, “ulcerative colitis”, “familial adenomatous polyposis”, “restorative proctocolectomy,” “ileoanal anastomosis” and “ileal pouches” was conducted. Two authors (GLC and EB) conducted two independent literature reviews, both using the same strategy. All articles from 1978 to December 2021 were included if they reported findings relating to dysplasia or cancer of the pouch and/or the ATZ and/or the rectal cuff (epidemiology, mechanisms, treatments, screening programme). Particular attention was paid to publications including endoscopic follow-up from which valuable conclusions could be drawn.

Additional articles were identified by cross-referencing papers from the initial search. The search excluded articles that were not in English and non-human studies, as well as editorials. Studies on patients suffering from Crohn’s diseases or indeterminate colitis have not been included in this review, given the rarity of the situation and the specific issues [33].

## 3. Dysplasia and Cancer of the Ileal Pouch in Patients with Ulcerative Colitis

### 3.1. Epidemiology and Risk Factors

According to the largest prospective study published, the incidence of ATZ dysplasia was estimated at 4.5% 10 years after surgery [34]. Several cases of adenocarcinoma of the rectal cuff and anastomosis have been reported since the 1990s [35,36,37,38,39,40,41,42]. However, the first case of ATZ adenocarcinoma to be described with anatomical precision was reported in 1997 by Sequens et al. [43]. Other cases have been reported since then [44,45,46,47,48,49,50,51,52,53,54]. The main published cohorts are summarised in Table 1.

The presence of dysplasia or cancer in the proctocolectomy specimen suggests that the patient is at risk of developing ATZ cancer [26,34,55,56]. Pedersen et al. described a case of ATZ adenocarcinoma following pouch excision without abdominoperineal excision [57]. RPC was originally carried out for high-grade rectal dysplasia. In view of the risk, total abdominoperineal excision should be discussed for patients requiring pouch removal. The duration of UC has also been identified as a risk factor for ATZ cancer, such as colorectal cancer [58]. Finally, no link has been established between the risk of ATZ dysplasia/cancer and the type of anastomosis performed (hand-sewn with mucosectomy/stapled) [42].

**Table 1:** Main published studies estimating the risk of ATZ dysplasia and cancer in patients with ulcerative colitis, including clinical and endoscopic follow-up.

Author, Year, [Ref.]	Design	Location of the Study	Pre-Op Colonic Dysplasia/Cancer	Number of Patients	Mean Follow-Up (Months)	Number of Cases	Identified Risk Factors
Tsunoda et al., 1990 [26]	Retrospective	England	10.2% of dysplasia, 6.8% of cancer	118	NA	3 dysplasia	Duration of the disease > 10 years Pre-op cancer
Schmitt et al., 1992 [59]	Prospective	USA	NA	50	8.6	0 dysplasia, 0 cancer	none
Ziv et al., 1994 [55]	Retrospective	USA	9.4% high-grade dysplasia, 4.3% cancer	254	28	8 low-grade dysplasia	Pre-op dysplasia or cancer
Haray et al., 1996 [60]	Retrospective	USA	1 patient with dysplasia	109	31	0 dysplasia, 0 cancer	none
Sarigol et al., 1999 [61]	Prospective	USA	6.6% dysplasia	76	60	0 dysplasia	none
O’Riordain et al., 2000 [56]	Retrospective	USA	10.5% dysplasia, 4.3% cancer	210	77	6 low-grade dysplasia, 1 high-grade dysplasia	Pre-op dysplasia or cancer
Remzi et al., 2003 [34]	Prospective	USA	14.6% dysplasia, 3.4% cancer	178	130	6 low-grade dysplasia, 2 high-grade dysplasia	Pre-op dysplasia or cancer
Kayaalp et al., 2003 [62]	Retrospective	Turkey	9.1% dysplasia	44	42	1 dysplasia	none
Kariv et al., 2010 [63]	Retrospective	USA	13.7% dysplasia, 1.8% cancer	3203	99	16 dysplasia, 10 cancers	Pre-op dysplasia or cancer
Mathis et al., 2011 [64]	Retrospective	USA	47% dysplasia, 9% cancer	100	71	1 adenocarcinoma	none
Zhu et al., 2013 [65]	Retrospective	USA	13.8% dysplasia, 4.3% cancer	123	89	1 indeterminate for dysplasia	none
Silva-Velazco et al., 2014 [66]	Retrospective	USA	10.5% dysplasia, 2.5% cancer	285	125	6 low-grade dysplasia, 3 high-grade dysplasia	Pre-op dysplasia or cancer
Block et al., 2015 [67]	Prospective	Sweden	46% low-grade dysplasia, 34% high-grade dysplasia, 20% cancer	56	216	4 indefinite for dysplasia	none
Lightner et al., 2020 [68]	Retrospective	USA	NA	3672	NA	7 low-grade dysplasia, 4 cancers	none

NA: not available; Pre-op: pre-operative.

Ravitch gave the first description of adenocarcinoma of the pouch, per se, following RPC for UC in 1984 [69]. Thirteen case reports have been published since then [70,71,72,73,74,75,76,77,78,79,80,81,82]. The main cohorts reported on are summarised in Table 2. The largest study estimated the risk of developing pouch cancer to be 0.2%, 0.4%, 0.8%, 2.4% and 3.4% 5, 10, 15, 20 and 25 years post-RPC, respectively, [63]. The cumulative incidence for pouch dysplasia at 5, 10, 15, 20 and 25 years was 0.8%, 1.3%, 1.5%, 2.2% and 3.2%, respectively.

The only risk factor identified for pouch cancer in this study was pre-operative colorectal cancer (adjusted hazard ratio of 13.43; *p* < 0.001) and dysplasia (adjusted hazard ratio of 3.62; *p* = 0.002). A link with primary sclerosing cholangitis has been put forward as a risk factor for pouch dysplasia and cancer [83]. Similar to ATZ neoplasia, the duration of UC has also been described as a risk factor [84,85]. Some studies suggested that chronic pouchitis might be a risk factor for dysplasia onset [85,86]. However, the largest available cohorts showed no link between pouchitis and dysplasia (or cancer) of the pouch [29,63]. Finally, pre-operative backwash ileitis has been put forward as a risk factor for pouch dysplasia [72]. However, other studies found no link with pouch dysplasia [87].

**Table 2:** Main published studies estimating the risk of pouch dysplasia and cancer in patients with ulcerative colitis, including adequate clinical and endoscopic follow-up.

Author, Year, [Ref.]	Design	Location of the Study	Pre-Op Colonic Dysplasia/Cancer	Number of Patients	Mean Follow-Up (Months)	Number of Cases	Identified Risk Factors
Emblem et al., 1988 [88]	Prospective	Norway	10.5% low-grade dysplasia	19	>36	0 dysplasia	none
Setti Carraro et al., 1994 [89]	Retrospective	England	23.3% dysplasia	60	97	0 dysplasia	none
Gullberg et al., 1997 [86]	Prospective	Sweden	20% low-grade dysplasia, 12% high-grade dysplasia	25	54	4 low-grade dysplasia, 1 high-grade dysplasia	Severe villous atrophy
Sarigol et al., 1999 [61]	Prospective	USA	6.6% dysplasia	76	60	0 dysplasia	none
Ettorre et al., 2000 [90]	Prospective	France/Italy	NA	21	85	0 dysplasia	none
Tiainen et al., 2001 [91]	Retrospective	Finland	NA	36	118	0 dysplasia	none
Heuschen et al., 2001 [92]	Retrospective	Germany	7.2% dysplasia, 9.5% cancer	308	48	1 cancer	none
Thompson-Fawcett et al., 2001 [93]	Prospective	Canada	NA for dysplasia, 10.4% cancer	106	>91	1 low-grade dysplasia	none
Hultén et al., 2002 [94]	Retrospective	Sweden	NA	40	360	3 low-grade dysplasia	none
Herline et al., 2003 [95]	Retrospective	USA	NA	160	101	1 low-grade dysplasia	none
Ståhlberg et al., 2003 [83]	Prospective	Sweden	43.8% dysplasia	32	144	5 low-grade dysplasia, 1 high grade dysplasia	primary sclerosing cholangitis
Börjesson et al., 2004 [96]	Prospective	Sweden	0 cancer	45	192	2 low-grade dysplasia	none
Nilubol et al., 2007 [97]	Prospective	USA	15.9% dysplasia, 5.8% cancer	118	65	1 indeterminate for dysplasia	none
Zmora et al., 2009 [98]	Retrospective	Israel	8.6% dysplasia, 7.6% cancer	185	97	1 cancer	none
Kariv et al., 2010 [63]	Retrospective	USA	13.7% dysplasia, 1.8% cancer	3203	99	8 dysplasia, 3 cancers	Pre-op dysplasia or cancer
Al-Sukhni et al., 2010 [99]	Retrospective	Canada	72.8% dysplasia, 27.2% cancer	81	76	1 dysplasia, 1 cancer	none
Hernández et al., 2010 [100]	Prospective	Puerto Rico	26% dysplasia	38	12	1 low-grade dysplasia	none
Burdyński et al., 2011 [101]	Retrospective	Poland	NA	87	139	2 low-grade dysplasia, 1 high-grade dysplasia	none
Shuno et al., 2011 [102]	Retrospective	Japan	NA	68	64	1 low-grade dysplasia, 1 high-grade dysplasia	none
O’Riordain et al., 2012 [58]	Retrospective	Canada	NA	2010	161	0.0015% of cancer	none
Kuiper et al., 2012 [103]	Prospective	The Netherlands	27.3% low-grade dysplasia, 18.2% high-grade dysplasia, 25% cancer	44	103	2 low-grade dysplasia	none
Derikx et al., 2014 [29]	Retrospective	The Netherlands	9.4% dysplasia, 4.2% cancer	1200	100	8 low-grade dysplasia, 1 high-grade dysplasia, 16 cancers	Pre-op dysplasia or cancer
Imam et al., 2014 [104]	Retrospective	USA	41.5% low-grade dysplasia, 6.2% high-grade dysplasia, 10.8% cancer	65	72	1 low-grade dysplasia, 1 high-grade dysplasia, 1 cancer	primary sclerosing cholangitis
Bobkiewicz et al., 2015 [85]	Retrospective	Poland	20.3% low-grade dysplasia, 9.1% high-grade dysplasia, 1.8% cancer	276	118	5 low-grade dysplasia, 3 high-grade dysplasia, 1 cancer	Pre-op dysplasia or cancer, duration of UC, duration of follow-up, pouchitis
Block et al., 2015 [67]	Prospective	Sweden	46% low-grade dysplasia, 34% high-grade dysplasia, 20% cancer	56	216	20 indefinite for dysplasia, 1 low-grade dysplasia	None
Ishii et al., 2016 [105]	Retrospective	Japan	27% dysplasia or cancer	90	120	1 low-grade dysplasia, 1 cancer	None
Mark-Christensen et al., 2018 [106]	Retrospective	Denmark	1.1% cancer	1723	155	2 cancers	None
Lightnert et al., 2020 [68]	Retrospective	USA	NA	3672	NA	2 cancers	none

NA: not available; Pre-op: pre-operative.

More anecdotally, other pouch and ATZ malignancies have been reported such as squamous cell carcinoma [63,66,69,106,107,108,109,110], pouch lymphoma [29,63,99,111,112,113,114,115,116,117], carcinoid tumour of the pouch [118,119] and malignant melanoma [120]. Moreover, the Danish nationwide cohort found a higher risk of non-melanoma skin and hepatobiliary cancers in patients undergoing IPAA for UC than in the gender- and age-matched comparison cohort of patients from a national civil database [106]. These results might be explained by the exposition to thiopurines and the co-existence of primitive sclerosing cholangitis, respectively. Similarly, an increased risk of renal cell cancer was highlighted in another study [121]. The main references in this topic could be as follows: [29,34,55,63,68,72,103].

### 3.2. Underlying Mechanisms

The risk of dysplasia and cancer of the ATZ and rectal cuff have been postulated more frequently in the case of stapled IPAA. Yet, stapled anastomosis does not seem to increase the risk of rectal cuff and ATZ cancers [84]. Given the better functional outcomes, stapled anastomosis is the preferred option according to the European guidelines [5]. The latter also state that the maximum length of anorectal mucosa left between the dentate line and anastomosis should not exceed 2 cm in order to lower the risk of cuffitis, dysplasia and cancer. Moreover, despite rectal mucosectomy, remaining islets of rectal mucosa can be found in up to 20% of patients after RPC with hand-sewn anastomosis for UC [122,123]. These data partly explain the rate of ATZ dysplasia and cancer observed in the case of hand-sewn anastomosis [58].

Furthermore, the presence of ATZ dysplasia and adenocarcinoma at the time of RPC has also been reported [124,125,126]. Sagayama et al. reported an incidence of preoperative dysplasia of ATZ at 4.4% [126]. This illustrates that, in some cases, the dysplasia might precede the IPAA. The third neoplastic pathway described is through chronic inflammation of the ATZ, which can be detected in up to four out of five patients after RPC for UC [127,128]. Similar to colitis-related colorectal cancer, the risk of ATZ dysplasia and cancer might increase in the context of chronic inflammation.

Veress et al. pointed out that some patients undergo mucosal adaptation with chronic villous atrophy after RPC (described as “type C” mucosal adaptation) [129]. This atrophy seems to be due to chronic inflammation [92,130,131]. Histologically, this adaptation (also called “colonic metaplasia”) is characterised by villous atrophy, crypt hyperplasia, neutrophilic and eosinophilic inflammation and increased Paneth and Goblet cell counts [132,133]. The number of patients with this type of mucosal adaptations increases the longer the follow-up period [134,135]. Changes in expression mucins have also been reported with an increase in colonic sulphomucins associated with the degree of villous atrophy and chronic inflammation [136,137,138].

This severe villous atrophy with chronic inflammation is assumed to highlight the risk of dysplasia and DNA aneuploidy [86,139]. Primary sclerosing cholangitis has also been linked to chronic villous atrophy [83]. This might partially account for the assumed higher risk of dysplasia and cancer in this subgroup of patients. However, atrophic pouch metaplasia is not always followed by dysplasia onset [96].

Microbiota are involved in the colonic metaplasia of the ileal reservoir [140,141,142]. Strict anaerobic bacteria predominate in the pouch, whereas facultative species predominate in UC patients with terminal ileostomy [143,144]. This change does not appear to occur in the case of FAP [143,145]. In the study by Kuisma et al., a higher overall faecal anaerobic bacterial count was associated with chronic villous atrophy and colonic metaplasia [146]. To highlight the role of the microbiota in the development of pouch dysplasia, changes in the mucosal pattern of ileal pouches, characterised by villous atrophy, have been reported to start as early as 6 days to 6 weeks after ileostomy suppression [147,148]. Das et al. studied the mucosal morphology of the pouch of patients with indefinite diversion (without pouch excision) [149]. None of the 20 patients developed dysplasia or pouch cancer (mean follow-up of 3.6 years after ileal diversion).

Sulphate-reducing bacteria are associated with greater sulphomucin expression in pouches [143]. Sulphate-reducing bacteria may interfere with goblet cell differentiation and, as a result of hydrogen sulphide production, may trigger epithelial apoptosis [143,150]. The latter phenomenon is mediated by inhibition of butyrate oxidation by hydrogen sulphide which impairs its use by epithelial cells [151]. Compared to non-colectomised patients, the concentration of short-chain fatty acids seems similar in pouches, albeit with an increased acetate ratio [144,152].

Depleted levels of secondary bile acids (lithocholic acid and deoxycholic acid) have been found in the pouch of UC patients compared to FAP patients [153]. Sinha et al. have proven that secondary bile acids have the capacity to mitigate inflammation in murine colitis models [153]. Finally, increased TLR4 expression (a bacterial antigen receptor) has been highlighted in endoscopically normal pouches compared to normal ileum [154]. This over-expression might be one of the pathways from dysbiosis to luminal inflammation.

The underlying pathology might also be involved in the risk of pouch inflammation, dysplasia and cancer. Indeed, compared to FAP patients, it has been proven that pro-inflammatory cytokines (such as TNFα) and pro-apoptotic proteins are up-regulated in endoscopically normal pouches of UC patients [155,156]. Modulation of autophagy markers in the ileal pouch of UC patients has also been described (decreased Beclin-1 protein levels) [157]. This might explain the higher incidence of pouch inflammation observed in UC patients.

Genetic aberrations are assumed to occur during the metaplasia-dysplasia-cancer sequence of the pouch, similar to colorectal carcinogenesis. The only modification identified by Gullberg et al. was the loss of heterozygosity at chromosome 5q14-22 [158]. Controversy surrounds the role of p53. Coull et al. found no correlation between ATZ dysplasia and overexpression of p53 (affecting one in two patients) [159]. On the contrary, other studies have highlighted a link between over-expressed p53 and aneuploidy, dysplasia and pouch cancer [160,161]. Finally, alterations in miRNA expression (mostly up-regulated) have been described in the pouches of UC patients [162,163].

The main references on all these underlying mechanisms could be as follows: [86,122,123,129,137,143,146].

### 3.3. Endoscopic Monitoring

Endoscopically, dysplasia and cancer of the pouch or ATZ can present as polypoid or non-polypoid lesions (including flat and ulcerated lesions) [164]. Only low-level evidence studies have been conducted as part of the endoscopic monitoring programme given the low prevalence of such complications. Hence, pouch monitoring remains controversial [165]. However, international guidelines based on risk stratification are available.

**Figure 2:**
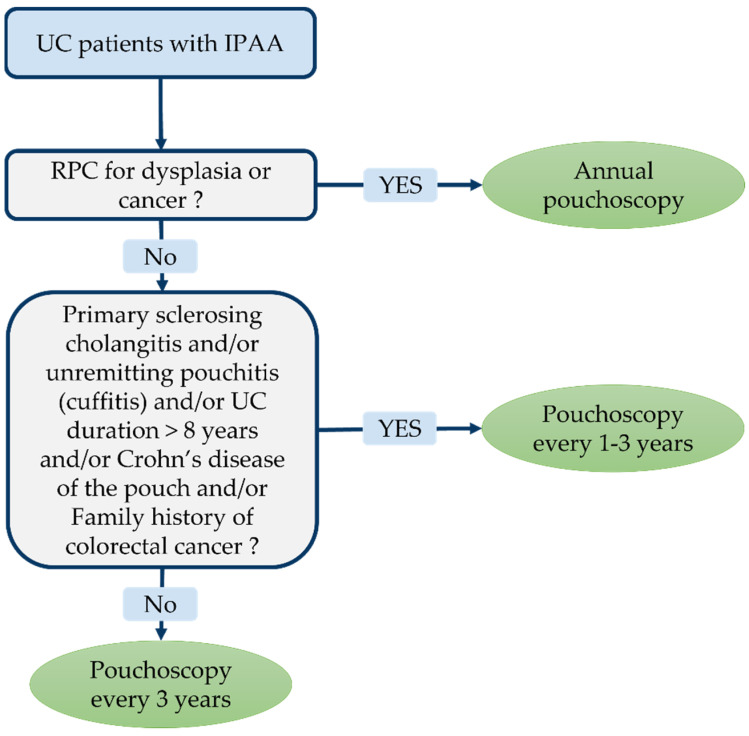
Endoscopic monitoring guidelines after restorative proctocolectomy (RPC) with ileal pouch-anal anastomosis (IPAA) for ulcerative colitis (UC) according the International Ileal Pouch Consortium [167,168].

The British Society of Gastroenterology (BSG) recommends annual pouchoscopy for high-risk asymptomatic patients, defined by the presence of at least one of the following criteria: type C mucosal changes, primary sclerosing cholangitis or RPC for dysplasia or cancer [166]. The European Crohn’s and Colitis Organisation (ECCO) also recommends annual pouchoscopy for high-risk patients (same criteria and unremitting pouchitis) [167,168]. Both societies recommend pouchoscopy every 5 years for low-risk patients (Figure 2). According to the American Society of Gastrointestinal Endoscopy (ASGE), annual monitoring is mandatory in the case of RPC for dysplasia or cancer, and may be considered for patients with type C mucosal changes, primary sclerosing cholangitis and refractory pouchitis [169]. Pouchoscopy is also indicated if the following symptoms develop, namely diarrhea, haematochezia, abdominal pain, iron deficiency or anemia, etc. Despite those recommendations, an American survey reported by Gu et al. found heterogeneous practices concerning the endoscopy interval [170]. A European retrospective cohort study found similar results with one-third of the cohort who had never undergone pouchoscopy during follow-up (median duration of 10.5 years) [171].

More recently, consensus guidelines on the diagnosis and classifications of ileal pouch disorders have been published by the International Ileal Pouch Consortium [172]. Experts recommend a surveillance pouchoscopy programme depending on individual risk. Annual pouchoscopy is advocated in the case of pre-colectomy diagnosis of colitis-related dysplasia or cancer. Pouchoscopy every 1–3 years is recommended for patients with associated primary sclerosing cholangitis, chronic pouchitis (or cuffitis), Crohn’s disease of the pouch, persistent ulcerative colitis (≥8 years) and in the event of a family history of colorectal cancer. If none of the afore-mentioned risk factors is present, pouchoscopy can be performed every 3 years.

**Figure 3:**
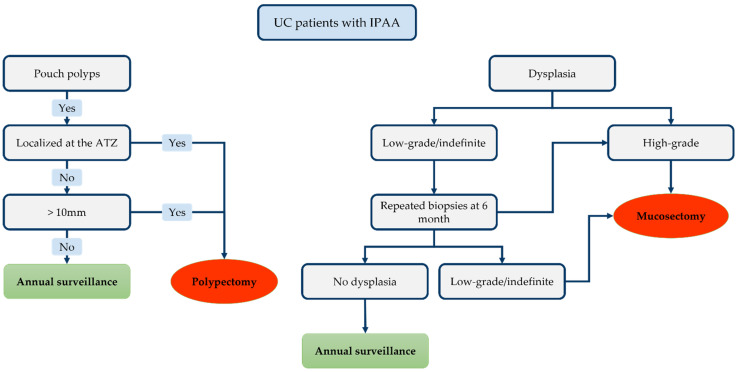
Proposed therapeutic algorithm for lesion detected on the ileal pouch-anal anastomosis (IPAA).

The therapeutic strategy is outlined in Figure 3. Although most of pouch polyps are inflammatory, polypectomy must be offered for pouch polyps exceeding 1 cm, for polyps localised on the ATZ/rectal cuff and symptomatic inflammatory polyps [173,174]. Repeated biopsies within 6 months might be advisable in case of low-grade dysplasia. Indeed, regression of low-grade dysplasia to normal mucosa has been reported in serial biopsies [30,34,55]. Regression has even been documented in high-grade dysplasia patients in some studies [29]. Such findings may be explained by sampling errors in an area where it is difficult to perform biopsies [175]. Therefore, multiple and repeated biopsies are mandatory on endoscopic examination of the pouch. Multiple biopsies (at least 3–4) must be taken from each of the following regions: the ATZ, pouch and afferent ileal limb [176,177]. The biopsies collected from each region must be analysed separately.

Moreover, Wu et al. suggested that a family history of colorectal cancer is associated with an increased risk of low-grade dysplasia progression [30]. Hence, such patients require increased vigilance. Finally, endoscopic mucosectomy is the preferred option for high-grade dysplasia and for recurrent (or persistently positive) low-grade dysplasia biopsies. The recent international consensus stated that patients with a history of dysplasia of the ATZ/rectal cuff (or pouch) must undergo close monitoring with early pouchoscopy 3–6 months after endoscopic treatment, and annually thereafter [172]. Complete surgical mucosectomy with pouch advancement (or pouch resection with terminal ileostomy) is indicated if endoscopic treatment fails or proves impossible.

The use of chromoendoscopy with targeted biopsies has been assessed in only one study which failed to highlight any benefits with this technique [103]. The use of high-magnification chromoscopic endoscopy has been recommended, but it has never been put to widespread use [178]. One way of improving the detection of cancer and dysplasia during endoscopy would be to develop innovative tools based on artificial intelligence [179].

The main references on endoscopic screening could be as follows: [166,167,169,172,176].

## 4. Adenomas, Dysplasia and Cancer of the ATZ and Ileal Pouch in Patients with FAP

### 4.1. Epidemiology and Risk Factors

The cumulative risk of developing ATZ adenoma has been estimated by Van Duijvendijk et al. as 8 and 18% at 3.5 and 7 years post-surgery, respectively, [180]. Tsunoda et al. had previously reported on a retrospective cohort of patients who developed ATZ dysplasia (high-grade for 21.4% of patients) [26]. Hoehner and Metcalf provided the first description of ATZ adenocarcinoma following RPC for FAP in 1994 [181]. Since then, seven case reports have been published [182,183,184,185,186,187,188]. The main cohorts highlighting the risk of dysplasia and cancer of the ATZ after restorative proctocolectomy for FAP are summarised in Table 3. Due to the low incidence of dysplasia and adenocarcinoma of the ATZ, no risk factors have been identified to date. However, stapled anastomosis appears to be linked to a higher risk of ATZ adenomas [18,23,180,189]. Severe colic disease (>1000 polyps) was identified as a risk factor in ATZ adenomas in one study [23].

Numerous cases of pouch adenomas following RPC for FAP have been reported [18,23,190,192,196,197,198,199,200,201,202,203,204,205,206]. This risk increases over time and prevalence has been estimated as 7%, 35% and 75% at 5, 10 and 15 years post-surgery, respectively, [207]. The presence of gastric adenoma, male gender and ≤ 18 years old at the time of surgery have been reported as risk factors for pouch adenomas by Ganschow et al. [208]. Goldstein et al. reported a higher incidence of pouch adenomas in the case of related duodenal adenomas [205]. On the contrary, Wu et al. found no correlation between the severity of duodenal disease (according to Spigelman’s classification system) and pouch adenomas [190]. Severe colic disease (>1000 polyps) has been identified by Tonelli et al. as a risk factor for pouch adenomas.

High-grade dysplasia and adenocarcinomas of the pouch have been documented in case reports [209,210,211,212,213,214,215,216,217]. The main cohorts highlighting the risk of dysplasia and cancer of the pouch after restorative proctocolectomy for FAP are summarised in Table 4. In terms of risk factors, no correlation has been established between pouchitis and the onset of dysplasia [193]. Data regarding the overall risk of pouch dysplasia and cancer post-IPAA and the impact of the anastomosis technique are sparse. The meta-analysis conducted by Lovegrove reported a lower rate of dysplasia with hand-sewn anastomosis (7.2% vs. 18.5% with double-stapled anastomosis). However, this result was not significant (*p* = 0.08) [218].

The main references on epidemiology and risk factor could be as follows: [23,180,194,196,207].

### 4.2. Underlying Mechanisms

The presence of residual rectal mucosa is one of the main reasons behind the onset of pouch adenomas in the case of stapled anastomosis [223]. On the other hand, remaining islets of rectal mucosa have been described in patients with hand-sewn anastomosis, despite mucosectomy [123].

Despite removal of the entire rectal mucosa, colonic metaplasia of the ileal mucosa was still observed, as in UC [224,225]. An increased rate of asymmetrical fission of the pouch crypts has been put forward to explain this hyperplasia [226]. Some authors assume that colonic metaplasia is due to faecal stasis in the pouch, which alters the luminal content and, consequently, adapts the epithelium [22,227]. Indeed, the number of post-IPAA faecal bacteria is 10 times higher than that reported following terminal ileostomy [228]. Functional changes have also been observed such as a change from mucins to colonic sulphomucins and the increased metabolism of primary conjugated bile acids [228,229,230]. To substantiate the theory of colonic metaplasia, cases of terminal ileostomy adenocarcinoma, in which the mucosa adjacent to the tumour presented colonic mucosa characteristics, have also been reported [231,232].

Friedrich et al. demonstrated that Glutathione S-transferase activity, which has a protective role in carcinogenesis, is lower in the pouch than in the proximal ileum [233]. Paiva et al. found fewer autophagy markers (ATG5 and MAP1LC3A) in the ileal pouch mucosa of FAP [157]. The heightened risk of adenoma is also due to the increased proliferation rate of epithelial cells in the pouch [234].

A molecular study by Will et al. provides data on the APC mutation spectrum in pouch adenomas [235]. In FAP, there is a correlation between germline and somatic APC mutation. In their study, Will et al. proved that pouch adenomas are genetically closer to colorectal adenomas. Recently, Kariv et al. described a link between pouch and cuff adenoma and the type and location of APC mutation [206]. The risk of adenoma was increased in the event of indel/deletion mutation and a higher number of adenomas per patient was linked to exon 15 mutation. Conversely, Groves et al. found no link between genotypic characteristics and pouch adenomas [200].

**Figure 4:**
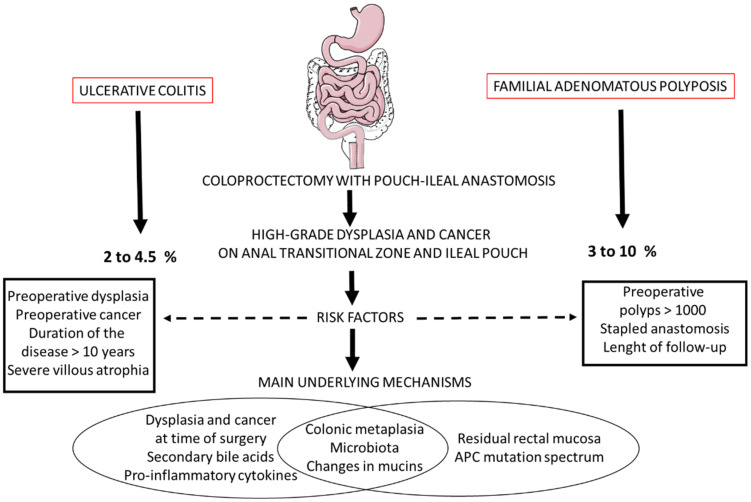
Main risk factors, frequency and putative underlying mechanisms of high-grade dysplasia and cancer in the anal transitional zone and ileal pouch following restorative coloproctectomy for ulcerative colitis and familial adenomatous polyposis.

Figure 4 shows the main risk factors, frequency and putative underlying mechanisms of high-grade dysplasia and cancer in the ATZ and ileal pouch following RPC for UC and FAP. The main references on all these underlying mechanisms could be as follows: [123,142,206,217]

### 4.3. Endoscopic Monitoring

**Figure 5:**
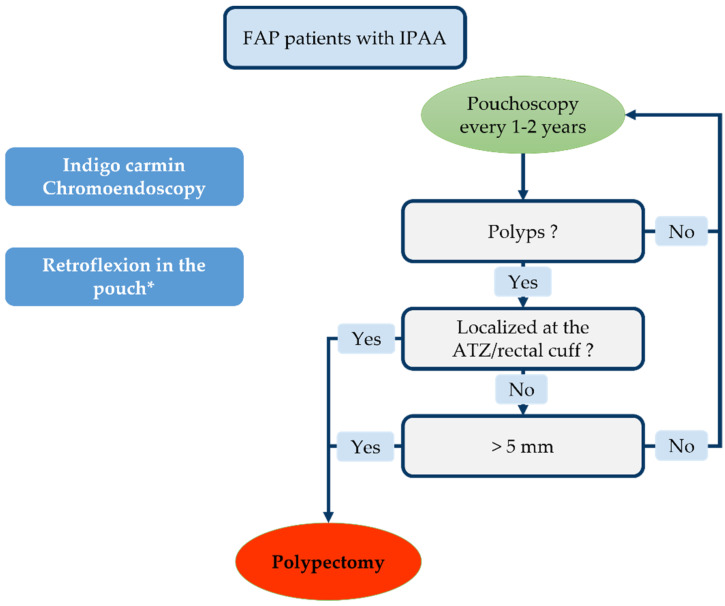
Endoscopic monitoring according to European Society of Gastrointestinal Endoscopy (ESGE) guidelines. * Depending on patient tolerance.

This high incidence of adenomas and reported cases of cancer of the pouch emphasize the need for endoscopic monitoring. The European Society of Gastrointestinal Endoscopy (ESGE) published guidelines in 2019 [236]. It advocates endoscopic pouch monitoring every 1–2 years in the case of FAP (Figure 5). The ESGE recommends removing pouch polyps > 5 mm and all ATZ and rectal cuff polyps. Cold snare polypectomy is the technique mostly used to remove pouch polyps [237]. All of these recommendations are sound but rely on a poor evidence base.

Some authors even advocate initiating thorough endoscopy monitoring 6 months and 1 year after surgery with 2 yearly follow-ups thereafter [238,239]. The use of indigo carmine chromoendoscopy promotes the detection of small adenomas (<5 mm) [219,240]. Retroflexion in the pouch has been seen to increase the adenoma detection rate in the cuff/ATZ without any specific complications [241]. Yet, retroflexion might be painful for patients not under general anesthesia; therefore, its use must be adapted to patients’ tolerance. Concerning bowel preparation, as for UC, a single sodium phosphate enema is usually sufficient to allow a complete examination of the pouch.

The major limiting factor in endoscopic monitoring is poor patient compliance. Douma et al. estimated that 8% of pouch patients fail to comply with recommended monitoring procedures [242] mainly due to lack of symptoms and the unpleasant nature of endoscopy per se. The main references on this endoscopic screening could be as follows: [236,239,241,242].

## 5. Discussion

We have collated available data on dysplasia and cancer of the ATZ and pouch in the case of UC and FAP. The overall incidence of pouch adenomas was assessed in up to 75% of patients 15 years post-surgery [207]. The risk of dysplasia and cancer is due to a combination of genetic predisposition (germline *APC* mutation), intestinal epithelial changes due to faecal stasis and the presence of residual rectal mucosa [227]. Smith et al. estimated that 75% of pouch-related cancers post-RPC with IPAA in FAP patients are located in the ATZ [27]. Particular attention must be given to this area during patient follow-up (digital examination of the area, systematic biopsies and a retroflexion view of the pouch).

The incidence rate of pouch adenocarcinoma has been estimated at 0.35% 20 years after surgery in UC patients [28]. The underlying mechanisms in the UC context are partly based on the chronic inflammation-dysplasia-cancer pathway [243]. Chronic exposure of the ileal mucosa to dysbiotic microbiota led to colonic type phenotypic changes through modulation of the short fatty acid chains available, mucin secretion and bile acid metabolism. Despite the low incidence, the poor prognosis of pouch adenocarcinoma (evidenced by a 30% mortality rate 1 year after cohort diagnosis reported by Kariv et al.) confirms the major benefit of regular pouchoscopy [63].

Our review has several limitations. First of all, many of the studies reported have retrospective, small sample size cohorts with heterogeneous follow-up. The heterogeneity of the surgical techniques used in the studies creates further bias [18,124,181,191]. Much progress has been made since 1988 and the first prospective cohort reported by Emblem et al., which explains the lack of evidence regarding one of the anastomotic techniques used (stapled vs. hand-sewn) [88]. In most of the studies focusing on the risk of adenoma, dysplasia and cancer of the IPAA, it is impossible to differentiate between adenomas located in the anal transitional zone and those in the rectal cuff or pouch [205,219,244]. The prevalence of ATZ lesions may well be under-estimated.

Some studies assessing the risk of pouch adenomas in FAP patients do not specify the presence and extent of dysplasia within the adenomas [190,192,194,195]. Yet, in the case of FAP, almost all of the adenomas led to dysplasia (unlike inflammatory polyps for UC), thereby emphasising the importance of estimating the incidence of pouch adenomas, regardless of the degree of dysplasia. The difficulty of the histological examination is yet another limitation in terms of assessing dysplasia of the ATZ and pouch. Hultén et al. reported on a significant disagreement between 2 expert pathologists in reporting low-grade dysplasia for the same 40 IPAA biopsies [94]. The complexity of the pathological analysis stems from the frequent presence of acute inflammation and regenerating epithelium, which interfere with the diagnosis of dysplasia [245]. The frequent use of “indefinite for dysplasia” by pathologists to characterise pouch biopsies also illustrates that difficulty [246]. This interobserver variability in diagnosing dysplasia might partly account for the low incidence reported. Moreover, the quality of data given to the pathologist by the endoscopist (location of the biopsies, clinical characteristics, endoscopic description of the pouch) and the biopsy specimens per se are frequently poor [247]. The introduction of a standardised endoscopic reporting template has been instrumental in improving those outcomes [248].

Finally, some cohorts reported not one case of dysplasia or cancer of the ATZ (or cuff or pouch) despite long-term follow-up [61,249,250]. The relative rarity of the event and the absence of a standardised biopsy protocol might explain these findings. It was also difficult to identify risk factors to guide the clinician and provide personalised patient follow-up because of the low incidence.

## 6. Conclusions

ATZ and ileal pouch cancers following RPC for FAP (or UC) are seldom detected in patients with IPAA. However, in view of the high mortality rate associated with this complication and given that pouchoscopy is a straightforward monitoring option, we recommend regular endoscopic monitoring for those patients. The development of endoscopic therapeutic options (less invasive than surgery) to remove dysplasia before cancer onset, further corroborates the need for a monitoring programme.

Additional studies are required to improve our knowledge of the underlying mechanisms (colonic metaplasia, dysbiosis) that perpetuate the emergence of dysplasia and cancer. A greater understanding of the factors involved may lead to preventive treatments (probiotics, anti-inflammatory drugs, etc.). This approach will help practitioners to identify those patients requiring follow-up and to initiate personalised endoscopic monitoring programmes.

## Figures and Tables

**Table 3:** Main published studies estimating the risk of adenomas, dysplasia and cancer of ATZ in patients with FAP including adequate clinical and endoscopic follow-up.

Author, Year, [Ref.]	Design	Location of the Study	Pre-Op Colonic Dysplasia/Cancer	Number of Patients	Median Follow-Up (Months)	Number of Cases	Identified Risk Factors
Tsunoda et al., 1990 [26]	Retrospective	England	100% of dysplasia, 28.6% of cancer	14	NA	12 dysplasia (3 high-grade)	none
Wu et al., 1998 [190]	Prospective	USA	NA	26	66	7 adenomas	none
Van Duijvendijk et al., 1999 [180]	Retrospective	The Netherlands	20.6% of cancer	97	66	13 adenomas (4 moderate dysplasia, 4 high-grade dysplasia)	Stapled anastomosis
Remzi et al., 2001 [23]	Retrospective	USA	NA	118	>42	27 adenomas, 1 cancer	Stapled anastomosis > 1000 polyps preoperatively
Ooi et al., 2003 [191]	Retrospective	USA	NA	148	NA	2 cancers	none
Von Roon et al., 2007 [189]	Retrospective	England	NA	140	123	52 adenomas (6 moderate dysplasia), 1 cancer	Stapled anastomosis
Booij et al., 2010 [192]	Retrospective	The Netherlands	77.8% of low-grade dysplasia, 11.1% of high-grade dysplasia, 11.1% of cancer	9	87	3 adenomas	none
Banasiewicz et al., 2011 [193]	Retrospective	Poland	NA	85	>24	19 low-grade dysplasia, 10 high-grade dysplasia, 1 cancer	none
Tonelli et al., 2012 [194]	Prospective	Italy	NA	69	133	3 adenomas	none
Wasmuth et al., 2013 [18]	Retrospective	Norway	NA	61	>164	18 adenomas, 1 cancer	Stapled anastomosis
Kennedy et al., 2014 [195]	Retrospective	USA	90% of low-grade dysplasia, 5% of high-grade dysplasia, 4.4% of cancer	95	91	9 adenomas	none
Lee et al., 2021 [196]	Retrospective	USA	17% of cancer	165	121	78 adenomas, 6 cancers	Stapled anastomosis

NA: not available.

**Table 4:** Main published studies estimating the risk of adenomas, dysplasia and cancer of the pouch in patients with FAP including adequate clinical and endoscopic follow-up.

Author, Year, [Ref.]	Design	Location of the Study	Pre-Op Colonic Dysplasia/Cancer	Number of Patients	Median Follow-Up (Months)	Number of Cases	Identified Risk Factors
Emblem et al., 1988 [88]	Prospective	Norway	23.1% of dysplasia, 23.1% of cancer	13	>36	10 adenomas (1 dysplasia)	none
Wu et al., 1998 [190]	Prospective	USA	NA	26	66	11 adenomas	none
Remzi et al., 2001 [23]	Retrospective	USA	NA	118	>42	23 adenomas	Stapled anastomosis
Groves et al., 2005 [200]	Prospective	England	NA	60	72	34 adenomas (23 low-grade dysplasia, 11 high-grade dysplasia)	Length of follow-up
Friederich et al., 2008 [219]	Retrospective	The Netherlands	NA	212	95	74 adenomas (25 high-grade dysplasia), 4 cancers	Stapled anastomosis
Campos et al., 2009 [220]	Retrospective	Brazil	60.2% of cancer	26	28	3 adenomas, 2 cancers	none
Booij et al., 2010 [192]	Retrospective	The Netherlands	77.8% of low-grade dysplasia, 11.1% of high-grade dysplasia, 11.1% of cancer	9	87	2 adenomas	none
Banasiewicz et al., 2011 [193]	Retrospective	Poland	NA	165	>24	13 low-grade dysplasia, 8 high-grade dysplasia, 5 cancers	none
Burdyński et al., 2011 [101]	Retrospective	Poland	NA	51	139	10 low-grade dysplasia, 5 high-grade dysplasia, 2 cancers	none
Tonelli et al., 2012 [194]	Prospective	Italy	NA	69	133	25 adenomas, 2 cancers	>50 years old at surgery >1000 polyps preoperatively
Wasmuth et al., 2013 [18]	Retrospective	Norway	NA	61	>164	14 adenomas	none
Boostrom et al., 2013 [221]	Retrospective	USA	NA	117	125	30 low-grade dysplasia, 1 cancer	None
Pommaret et al., 2013 [222]	Retrospective	France	NA	118	180	57 adenomas (7 high-grade dysplasia)	Duration of follow-up, advanced duodenal adenomas
Goldstein et al., 2015 [205]	Retrospective	Israel	NA	59	140	15 adenomas	Duodenal adenomas
Kariv et al., 2019 [206]	Retrospective	Israel	8.9% of high-grade dysplasia, 8.9% of cancer	45	>121	12 adenomas	Indel/deletion mutation of APC
Lee et al., 2021 [196]	Retrospective	USA	17% of cancer	165	121	47 adenomas	None

NA: not available.

## References

[1] McLaughlin S.D., Clark S.K., Tekkis P.P., Ciclitira P.J., Nicholls R.J. (2008). Review Article: Restorative Proctocolectomy, Indications, Management of Complications and Follow-up—A Guide for Gastroenterologists. Aliment. Pharm..

[2] Ungaro R., Mehandru S., Allen P.B., Peyrin-Biroulet L., Colombel J.-F. (2017). Ulcerative Colitis. Lancet.

[3] Ordás I., Eckmann L., Talamini M., Baumgart D.C., Sandborn W.J. (2012). Ulcerative Colitis. Lancet.

[4] Frolkis A.D., Dykeman J., Negrón M.E., Debruyn J., Jette N., Fiest K.M., Frolkis T., Barkema H.W., Rioux K.P., Panaccione R. (2013). Risk of Surgery for Inflammatory Bowel Diseases Has Decreased over Time: A Systematic Review and Meta-Analysis of Population-Based Studies. Gastroenterology.

[5] Magro F., Gionchetti P., Eliakim R., Ardizzone S., Armuzzi A., Barreiro-de Acosta M., Burisch J., Gecse K.B., Hart A.L., Hindryckx P. (2017). Third European Evidence-Based Consensus on Diagnosis and Management of Ulcerative Colitis. Part 1: Definitions, Diagnosis, Extra-Intestinal Manifestations, Pregnancy, Cancer Surveillance, Surgery, and Ileo-Anal Pouch Disorders. J. Crohns Colitis.

[6] Jasperson K.W., Tuohy T.M., Neklason D.W., Burt R.W. (2010). Hereditary and Familial Colon Cancer. Gastroenterology.

[7] Galiatsatos P., Foulkes W.D. (2006). Familial Adenomatous Polyposis. Am. J. Gastroenterol..

[8] Campos F.G. (2014). Surgical Treatment of Familial Adenomatous Polyposis: Dilemmas and Current Recommendations. World J. Gastroenterol..

[9] Trigui A., Frikha F., Rejab H., Ben Ameur H., Triki H., Ben Amar M., Mzali R. (2014). Ileal Pouch-Anal Anastomosis: Points of Controversy. J. Visc. Surg..

[10] Mège D., Figueiredo M.N., Manceau G., Maggiori L., Bouhnik Y., Panis Y. (2016). Three-Stage Laparoscopic Ileal Pouch-Anal Anastomosis Is the Best Approach for High-Risk Patients with Inflammatory Bowel Disease: An Analysis of 185 Consecutive Patients. J. Crohn’s Colitis.

[11] Francone T.D., Champagne B. (2013). Considerations and Complications in Patients Undergoing Ileal Pouch Anal Anastomosis. Surg. Clin. N. Am..

[12] Delaini G.G., Scaglia M., Colucci G., Hultén L. (2005). The Ileoanal Pouch Procedure in the Long-Term Perspective: A Critical Review. Tech. Coloproctol..

[13] Beyer-Berjot L., Maggiori L., Birnbaum D., Lefevre J.H., Berdah S., Panis Y. (2013). A Total Laparoscopic Approach Reduces the Infertility Rate After Ileal Pouch-Anal Anastomosis: A 2-Center Study. Ann. Surg..

[14] Fleming F.J., Francone T.D., Kim M.J., Gunzler D., Messing S., Monson J.R.T. (2011). A Laparoscopic Approach Does Reduce Short-Term Complications in Patients Undergoing Ileal Pouch-Anal Anastomosis. Dis. Colon Rectum.

[15] Ng K.-S., Gonsalves S.J., Sagar P.M. (2019). Ileal-Anal Pouches: A Review of Its History, Indications, and Complications. World J. Gastroenterol..

[16] Régimbeau J.M., Panis Y., Pocard M., Hautefeuille P., Valleur P. (2001). Handsewn Ileal Pouch-Anal Anastomosis on the Dentate Line after Total Proctectomy: Technique to Avoid Incomplete Mucosectomy and the Need for Long-Term Follow-up of the Anal Transition Zone. Dis. Colon Rectum.

[17] Parks A.G., Nicholls R.J. (1978). Proctocolectomy without Ileostomy for Ulcerative Colitis. Br. Med. J..

[18] Wasmuth H.H., Tranø G., Myrvold H.E., Aabakken L., Bakka A. (2013). Adenoma Formation and Malignancy after Restorative Proctocolectomy with or without Mucosectomy in Patients with Familial Adenomatous Polyposis. Dis. Colon Rectum.

[19] Thompson-Fawcett M.W., Warren B.F., Mortensen N.J. (1998). A New Look at the Anal Transitional Zone with Reference to Restorative Proctocolectomy and the Columnar Cuff. Br. J. Surg..

[20] Holder-Murray J., Fichera A. (2009). Anal Transition Zone in the Surgical Management of Ulcerative Colitis. World J. Gastroenterol..

[21] Thompson-Fawcett M.W., Mortensen N.J. (1996). Anal Transitional Zone and Columnar Cuff in Restorative Proctocolectomy. Br. J. Surg..

[22] Chambers W.M., McC Mortensen N.J. (2007). Should Ileal Pouch-Anal Anastomosis Include Mucosectomy?. Colorectal Dis..

[23] Remzi F.H., Church J.M., Bast J., Lavery I.C., Strong S.A., Hull T.L., Harris G.J., Delaney C.P., O’Riordain M.G., McGannon E.A. (2001). Mucosectomy vs. Stapled Ileal Pouch-Anal Anastomosis in Patients with Familial Adenomatous Polyposis: Functional Outcome and Neoplasia Control. Dis. Colon Rectum.

[24] Kirat H.T., Remzi F.H., Kiran R.P., Fazio V.W. (2009). Comparison of Outcomes after Hand-Sewn versus Stapled Ileal Pouch-Anal Anastomosis in 3109 Patients. Surgery.

[25] Scarpa M., van Koperen P.J., Ubbink D.T., Hommes D.W., Ten Kate F.J.W., Bemelman W.A. (2007). Systematic Review of Dysplasia after Restorative Proctocolectomy for Ulcerative Colitis. Br. J. Surg..

[26] Tsunoda A., Talbot I.C., Nicholls R.J. (1990). Incidence of Dysplasia in the Anorectal Mucosa in Patients Having Restorative Proctocolectomy. Br. J. Surg..

[27] Smith J.C., Schäffer M.W., Ballard B.R., Smoot D.T., Herline A.J., Adunyah S.E., M’Koma A.E. (2013). Adenocarcinomas After Prophylactic Surgery For Familial Adenomatous Polyposis. J. Cancer Ther..

[28] Selvaggi F., Pellino G., Canonico S., Sciaudone G. (2014). Systematic Review of Cuff and Pouch Cancer in Patients with Ileal Pelvic Pouch for Ulcerative Colitis. Inflamm. Bowel Dis..

[29] Derikx L.A.A.P., Kievit W., Drenth J.P.H., de Jong D.J., Ponsioen C.Y., Oldenburg B., van der Meulen-de Jong A.E., Dijkstra G., Grubben M.J.A.L., van Laarhoven C.J.H.M. (2014). Prior Colorectal Neoplasia Is Associated with Increased Risk of Ileoanal Pouch Neoplasia in Patients with Inflammatory Bowel Disease. Gastroenterology.

[30] Wu X.-R., Remzi F.H., Liu X.-L., Lian L., Stocchi L., Ashburn J., Shen B. (2014). Disease Course and Management Strategy of Pouch Neoplasia in Patients with Underlying Inflammatory Bowel Diseases. Inflamm. Bowel Dis..

[31] Sansone S., Nakajima T., Saito Y. (2017). Endoscopic Submucosal Dissection of a Large Neoplastic Lesion at the Ileorectal Anastomosis in a Familial Adenomatous Polyposis Patient. Dig. Endosc..

[32] Sugimoto T., Yoichi T., Suzuki K., Kawai T., Yashima Y., Sato S., Kawamoto J., Obi S. (2014). Endoscopic Submucosal Dissection to Treat Ileal High-Grade Dysplasia after Ileoanal Anastomosis for Familial Adenomatous Polyposis: Report of a Case. Clin. J. Gastroenterol..

[33] Adamina M., Bonovas S., Raine T., Spinelli A., Warusavitarne J., Armuzzi A., Bachmann O., Bager P., Biancone L., Bokemeyer B. (2020). ECCO Guidelines on Therapeutics in Crohn’s Disease: Surgical Treatment. J. Crohn’s Colitis.

[34] Remzi F.H., Fazio V.W., Delaney C.P., Preen M., Ormsby A., Bast J., O’Riordain M.G., Strong S.A., Church J.M., Petras R.E. (2003). Dysplasia of the Anal Transitional Zone after Ileal Pouch-Anal Anastomosis: Results of Prospective Evaluation after a Minimum of Ten Years. Dis. Colon Rectum.

[35] Stern H., Walfisch S., Mullen B., McLeod R., Cohen Z. (1990). Cancer in an Ileoanal Reservoir: A New Late Complication?. Gut.

[36] Puthu D., Rajan N., Rao R., Rao L., Venugopal P. (1992). Carcinoma of the Rectal Pouch Following Restorative Proctocolectomy. Report of a Case. Dis. Colon Rectum.

[37] Rodriguez-Sanjuan J.C., Polavieja M.G., Naranjo A., Castillo J. (1995). Adenocarcinoma in an Ileal Pouch for Ulcerative Colitis. Dis. Colon Rectum.

[38] Hyman N. (2002). Rectal Cancer as a Complication of Stapled IPAA. Inflamm. Bowel Dis..

[39] Negi S.S., Chaudhary A., Gondal R. (2003). Carcinoma of Pelvic Pouch Following Restorative Proctocolectomy: Report of a Case and Review of the Literature. Dig. Surg..

[40] Lee S.W., Sonoda T., Milsom J.W. (2005). Three Cases of Adenocarcinoma Following Restorative Proctocolectomy with Hand-Sewn Anastomosis for Ulcerative Colitis: A Review of Reported Cases in the Literature. Colorectal Dis..

[41] Gerich M.E., McManus M.C., McCarter M., Fukami N. (2011). Multifocal Pouch Body Adenocarcinoma Following Ileal Pouch-Anal Anastomosis (IPAA) for Ulcerative Colitis. Inflamm. Bowel Dis..

[42] Das P., Johnson M.W., Tekkis P.P., Nicholls R.J. (2007). Risk of Dysplasia and Adenocarcinoma Following Restorative Proctocolectomy for Ulcerative Colitis. Colorectal Dis..

[43] Sequens R. (1997). Cancer in the Anal Canal (Transitional Zone) after Restorative Proctocolectomy with Stapled Ileal Pouch-Anal Anastomosis. Int. J. Colorectal Dis..

[44] Baratsis S., Hadjidimitriou F., Christodoulou M., Lariou K. (2002). Adenocarcinoma in the Anal Canal after Ileal Pouch-Anal Anastomosis for Ulcerative Colitis Using a Double Stapling Technique: Report of a Case. Dis. Colon Rectum.

[45] Laureti S., Ugolini F., D’Errico A., Rago S., Poggioli G. (2002). Adenocarcinoma below Ileoanal Anastomosis for Ulcerative Colitis: Report of a Case and Review of the Literature. Dis. Colon Rectum.

[46] Rotholtz N.A., Pikarsky A.J., Singh J.J., Wexner S.D. (2001). Adenocarcinoma Arising from along the Rectal Stump after Double-Stapled Ileorectal J-Pouch in a Patient with Ulcerative Colitis: The Need to Perform a Distal Anastomosis. Report of a Case. Dis. Colon Rectum.

[47] Bell S.W., Parry B., Neill M. (2003). Adenocarcinoma in the Anal Transitional Zone after Ileal Pouch for Ulcerative Colitis: Report of a Case. Dis. Colon Rectum.

[48] Sagar P. (2006). Adenocarcinoma in a Pouch without a Preceeding History of Dysplasia. Colorectal Dis..

[49] Ota H., Yamazaki K., Endoh W., Hojo S., Fukunaga H., Yoshioka S., Okada Y., Okamoto S., Ueda N., Maeura Y. (2007). Adenocarcinoma Arising below an Ileoanal Anastomosis after Restorative Proctocolectomy for Ulcerative Colitis: Report of a Case. Surg. Today.

[50] Ruffolo C., Scarpa M., Polese L., Angriman I. (2007). Adenocarcinoma after Restorative Proctocolectomy for Cancer in Ulcerative Colitis. Int. J. Colorectal Dis..

[51] Chia C.S., Chew M.H., Chau Y.P., Eu K.W., Ho K.S. (2008). Adenocarcinoma of the Anal Transitional Zone after Double Stapled Ileal Pouch-Anal Anastomosis for Ulcerative Colitis. Colorectal Dis..

[52] Panier-Suffat L., Marracino M., Resegotti A., Astegiano M., Giustetto A., Garino M., Pellicano R., Fronda G. (2009). Anal Transitional Zone Adenocarcinoma Following Restorative Proctocolectomy for Ulcerative Colitis: Case Report and Review of Literature. Acta Gastroenterol. Belg..

[53] Alessandroni L., Kohn A., Capaldi M., Guadagni I., Scotti A., Tersigni R. (2012). Adenocarcinoma below Stapled Ileoanal Anastomosis after Restorative Proctocolectomy for Ulcerative Colitis. Updates Surg..

[54] Morelli L., Luca M., Palmeri M., Matteo P., Tartaglia D., Dario T., Guadagni S., Simone G., Di Candio G., Giulio D.C. (2014). Adenocarcinoma on J-Pouch after Proctocolectomy for Ulcerative Colitis-Case Report and Review of Literature. Int. J. Colorectal Dis..

[55] Ziv Y., Fazio V.W., Sirimarco M.T., Lavery I.C., Goldblum J.R., Petras R.E. (1994). Incidence, Risk Factors, and Treatment of Dysplasia in the Anal Transitional Zone after Ileal Pouch-Anal Anastomosis. Dis. Colon Rectum.

[56] O’Riordain M.G., Fazio V.W., Lavery I.C., Remzi F., Fabbri N., Meneu J., Goldblum J., Petras R.E. (2000). Incidence and Natural History of Dysplasia of the Anal Transitional Zone after Ileal Pouch-Anal Anastomosis: Results of a Five-Year to Ten-Year Follow-Up. Dis. Colon Rectum.

[57] Pedersen M.E., Rahr H.B., Fenger C., Qvist N. (2008). Adenocarcinoma Arising from the Rectal Stump Eleven Years after Excision of an Ileal J-Pouch in a Patient with Ulcerative Colitis: Report of a Case. Dis. Colon Rectum.

[58] O’Riordan J.M., Kirsch R., Mohseni M., McLeod R.S., Cohen Z. (2012). Long-Term Risk of Adenocarcinoma Post-Ileal Pouch-Anal Anastomosis for Ulcerative Colitis: Report of Two Cases and Review of the Literature. Int. J. Colorectal Dis..

[59] Schmitt S.L., Wexner S.D., Lucas F.V., James K., Nogueras J.J., Jagelman D.G. (1992). Retained Mucosa after Double-Stapled Ileal Reservoir and Ileoanal Anastomosis. Dis. Colon Rectum.

[60] Haray P.N., Amarnath B., Weiss E.G., Nogueras J.J., Wexner S.D. (1996). Low Malignant Potential of the Double-Stapled Ileal Pouch-Anal Anastomosis. Br. J. Surg..

[61] Sarigol S., Wyllie R., Gramlich T., Alexander F., Fazio V., Kay M., Mahajan L. (1999). Incidence of Dysplasia in Pelvic Pouches in Pediatric Patients after Ileal Pouch-Anal Anastomosis for Ulcerative Colitis. J. Pediatr. Gastroenterol. Nutr..

[62] Kayaalp C., Nessar G., Akoglu M., Atalay F. (2003). Elimination of Mucosectomy during Restorative Proctocolectomy in Patients with Ulcerative Colitis May Provide Better Results in Low-Volume Centers. Am. J. Surg..

[63] Kariv R., Remzi F.H., Lian L., Bennett A.E., Kiran R.P., Kariv Y., Fazio V.W., Lavery I.C., Shen B. (2010). Preoperative Colorectal Neoplasia Increases Risk for Pouch Neoplasia in Patients with Restorative Proctocolectomy. Gastroenterology.

[64] Mathis K.L., Benavente-Chenhalls L.A., Dozois E.J., Wolff B.G., Larson D.W. (2011). Short- and Long-Term Surgical Outcomes in Patients Undergoing Proctocolectomy with Ileal Pouch-Anal Anastomosis in the Setting of Primary Sclerosing Cholangitis. Dis. Colon Rectum.

[65] Zhu H., Wu X., Queener E., Kiran R.P., Remzi F.H., Shen B. (2013). Clinical Value of Surveillance Pouchoscopy in Asymptomatic Ileal Pouch Patients with Underlying Inflammatory Bowel Disease. Surg. Endosc..

[66] Silva-Velazco J., Stocchi L., Wu X., Shen B., Remzi F.H. (2014). Twenty-Year-Old Stapled Pouches for Ulcerative Colitis without Evidence of Rectal Cancer: Implications for Surveillance Strategy?. Dis. Colon Rectum.

[67] Block M., Börjesson L., Willén R., Bengtson J., Lindholm E., Brevinge H., Saksena P. (2015). Neoplasia in the Colorectal Specimens of Patients with Ulcerative Colitis and Ileal Pouch-Anal Anastomosis—Need for Routine Surveillance?. Scand. J. Gastroenterol..

[68] Lightner A.L., Vaidya P., Vogler S., McMichael J., Jia X., Regueiro M., Qazi T., Steele S.R., Church J. (2020). Surveillance Pouchoscopy for Dysplasia: Cleveland Clinic Ileoanal Pouch Anastomosis Database. Br. J. Surg..

[69] Ravitch M.M. (1984). The Reception of New Operations. Ann. Surg..

[70] Vieth M., Grunewald M., Niemeyer C., Stolte M. (1998). Adenocarcinoma in an Ileal Pouch after Prior Proctocolectomy for Carcinoma in a Patient with Ulcerative Pancolitis. Virchows Arch..

[71] Iwama T., Kamikawa J., Higuchi T., Yagi K., Matsuzaki T., Kanno J., Maekawa A. (2000). Development of Invasive Adenocarcinoma in a Long-Standing Diverted Ileal J-Pouch for Ulcerative Colitis: Report of a Case. Dis. Colon Rectum.

[72] Heuschen U.A., Heuschen G., Autschbach F., Allemeyer E.H., Herfarth C. (2001). Adenocarcinoma in the Ileal Pouch: Late Risk of Cancer after Restorative Proctocolectomy. Int. J. Colorectal Dis..

[73] Bentrem D.J., Wang K.L., Stryker S.J. (2003). Adenocarcinoma in an Ileal Pouch Occurring 14 Years after Restorative Proctocolectomy: Report of a Case. Dis. Colon Rectum.

[74] Hassan C., Zullo A., Speziale G., Stella F., Lorenzetti R., Morini S. (2003). Adenocarcinoma of the Ileoanal Pouch Anastomosis: An Emerging Complication?. Int. J. Colorectal Dis..

[75] Knupper N., Straub E., Terpe H.J., Vestweber K.H. (2006). Adenocarcinoma of the Ileoanal Pouch for Ulcerative Colitis—A Complication of Severe Chronic Atrophic Pouchitis?. Int. J. Colorectal Dis..

[76] Walker M., Radley S. (2006). Adenocarcinoma in an Ileoanal Pouch Formed for Ulcerative Colitis in a Patient with Primary Sclerosing Cholangitis and a Liver Transplant: Report of a Case and Review of the Literature. Dis. Colon Rectum.

[77] Naik V.S., Patil S.B., Scholefield J., Kaye P.V., James P.D., Ilyas M., Zaitoun A.M. (2008). Adenocarcinoma Arising in a Background of Chronic Atrophic Pouchitis in an Ileoanal Pouch for Ulcerative Colitis. Histopathology.

[78] Ault G.T., Nunoo-Mensah J.W., Johnson L., Vukasin P., Kaiser A., Beart R.W. (2009). Adenocarcinoma Arising in the Middle of Ileoanal Pouches: Report of Five Cases. Dis. Colon Rectum.

[79] Marmorale C., Stortoni P., Siquini W., Scartozzi M., Berardi R., Mandolesi A., Bearzi I., Fianchini A. (2011). Adenocarcinoma Arising from Ileoanal J-Pouch Mucosa: An Announced Event?. Inflamm. Bowel Dis..

[80] Branco B.C., Sachar D.B., Heimann T., Sarpel U., Harpaz N., Greenstein A.J. (2009). Adenocarcinoma Complicating Restorative Proctocolectomy for Ulcerative Colitis with Mucosectomy Performed by Cavitron Ultrasonic Surgical Aspirator. Colorectal Dis..

[81] Koh P.K., Doumit J., Downs-Kelly E., Bronner M.P., Salimi R., Fazio V.W., Vogel J.D. (2008). Ileo-Anal j-Pouch Cancer: An Unusual Case in an Unusual Location. Tech. Coloproctol..

[82] O’Mahoney P.R.A., Scherl E.J., Lee S.W., Milsom J.W. (2015). Adenocarcinoma of the Ileal Pouch Mucosa: Case Report and Literature Review. Int. J. Colorectal Dis..

[83] Ståhlberg D., Veress B., Tribukait B., Broomé U. (2003). Atrophy and Neoplastic Transformation of the Ileal Pouch Mucosa in Patients with Ulcerative Colitis and Primary Sclerosing Cholangitis: A Case Control Study. Dis. Colon Rectum.

[84] M’Koma A.E., Moses H.L., Adunyah S.E. (2011). Inflammatory Bowel Disease-Associated Colorectal Cancer: Proctocolectomy and Mucosectomy Do Not Necessarily Eliminate Pouch-Related Cancer Incidences. Int. J. Colorectal Dis..

[85] Bobkiewicz A., Krokowicz L., Paszkowski J., Studniarek A., Szmyt K., Majewski J., Walkowiak J., Majewski P., Drews M., Banasiewicz T. (2015). Large Bowel Mucosal Neoplasia in the Original Specimen May Increase the Risk of Ileal Pouch Neoplasia in Patients Following Restorative Proctocolectomy for Ulcerative Colitis. Int. J. Colorectal Dis..

[86] Gullberg K., Ståhlberg D., Liljeqvist L., Tribukait B., Reinholt F.P., Veress B., Löfberg R. (1997). Neoplastic Transformation of the Pelvic Pouch Mucosa in Patients with Ulcerative Colitis. Gastroenterology.

[87] Haskell H., Andrews C.W., Reddy S.I., Dendrinos K., Farraye F.A., Stucchi A.F., Becker J.M., Odze R.D. (2005). Pathologic Features and Clinical Significance of “Backwash” Ileitis in Ulcerative Colitis. Am. J. Surg. Pathol..

[88] Emblem R., Bergan A., Larsen S. (1988). Straight Ileoanal Anastomosis with Preserved Anal Mucosa for Ulcerative Colitis and Familial Polyposis. Scand. J. Gastroenterol..

[89] Setti Carraro P., Talbot I.C., Nicholls R.J. (1994). Longterm Appraisal of the Histological Appearances of the Ileal Reservoir Mucosa after Restorative Proctocolectomy for Ulcerative Colitis. Gut.

[90] Ettorre G.M., Pescatori M., Panis Y., Nemeth J., Crescenzi A., Valleur P. (2000). Mucosal Changes in Ileal Pouches after Restorative Proctocolectomy for Ulcerative and Crohn’s Colitis. Dis. Colon Rectum.

[91] Tiainen J., Matikainen M., Aitola P., Hiltunen K.M., Mattila J. (2001). Histological and Macroscopic Changes in the Pelvic Pouch: Long-Term Follow up after Restorative Proctocolectomy for Ulcerative Colitis (UC). Colorectal Dis..

[92] Heuschen U.A., Autschbach F., Allemeyer E.H., Zöllinger A.M., Heuschen G., Uehlein T., Herfarth C., Stern J. (2001). Long-Term Follow-up after Ileoanal Pouch Procedure: Algorithm for Diagnosis, Classification, and Management of Pouchitis. Dis. Colon Rectum.

[93] Thompson-Fawcett M.W., Marcus V., Redston M., Cohen Z., McLeod R.S. (2001). Risk of Dysplasia in Long-Term Ileal Pouches and Pouches with Chronic Pouchitis. Gastroenterology.

[94] Hultén L., Willén R., Nilsson O., Safarani N., Haboubi N. (2002). Mucosal Assessment for Dysplasia and Cancer in the Ileal Pouch Mucosa in Patients Operated on for Ulcerative Colitis--a 30-Year Follow-up Study. Dis. Colon Rectum.

[95] Herline A.J., Meisinger L.L., Rusin L.C., Roberts P.L., Murray J.J., Coller J.A., Marcello P.W., Schoetz D.J. (2003). Is Routine Pouch Surveillance for Dysplasia Indicated for Ileoanal Pouches?. Dis. Colon Rectum.

[96] Börjesson L., Willén R., Haboubi N., Duff S.E., Hultén L. (2004). The Risk of Dysplasia and Cancer in the Ileal Pouch Mucosa after Restorative Proctocolectomy for Ulcerative Proctocolitis Is Low: A Long-Term Term Follow-up Study. Colorectal Dis..

[97] Nilubol N., Scherl E., Bub D.S., Gorfine S.R., Marion J., Harris M.T., Kornbluth A., Lichtiger S., Rubin P., George J. (2007). Mucosal Dysplasia in Ileal Pelvic Pouches after Restorative Proctocolectomy. Dis. Colon Rectum.

[98] Zmora O., Spector D., Dotan I., Klausner J.M., Rabau M., Tulchinsky H. (2009). Is Stapled Ileal Pouch Anal Anastomosis a Safe Option in Ulcerative Colitis Patients with Dysplasia or Cancer?. Int. J. Colorectal Dis..

[99] Al-Sukhni W., McLeod R.S., MacRae H., O’Connor B., Huang H., Cohen Z. (2010). Oncologic Outcome in Patients with Ulcerative Colitis Associated with Dyplasia or Cancer Who Underwent Stapled or Handsewn Ileal Pouch-Anal Anastomosis. Dis. Colon Rectum.

[100] Hernández J.D.M., Jiménez-Huyke C., Rosado K., González-Keelan C., Lojo J.J., Torres E.A. (2010). Surveillance for Dysplasia in Patients with Ileal Pouch-Anal Anastomosis for Ulcerative Colitis: An Interim Analysis. Dig. Dis. Sci..

[101] Burdyński R., Banasiewicz T., Marciniak R., Biczysko M., Szmeja J., Paszkowski J., Grochowalski M., Maik J., Majewski P., Krokowicz P. (2011). Intestinal Pouch Complications in Patients Who Underwent Restorative Proctocolectomy for Ulcerative Colitis and Familial Adenomatous Polyposis in 1985–2008. Pol. Przegl. Chir..

[102] Shuno Y., Hata K., Sunami E., Shinozaki M., Kawai K., Kojima T., Tsurita G., Hiyoshi M., Tsuno N.H., Kitayama J. (2011). Is Surveillance Endoscopy Necessary after Colectomy in Ulcerative Colitis?. ISRN Gastroenterol..

[103] Kuiper T., Vlug M.S., van den Broek F.J.C., Tytgat K.M.a.J., van Eeden S., Fockens P., Bemelman W.A., Dekker E. (2012). The Prevalence of Dysplasia in the Ileoanal Pouch Following Restorative Proctocolectomy for Ulcerative Colitis with Associated Dysplasia. Colorectal Dis..

[104] Imam M.H., Eaton J.E., Puckett J.S., Loftus E.V., Mathis K.L., Gossard A.A., Talwalkar J.A., Lindor K.D. (2014). Neoplasia in the Ileoanal Pouch Following Colectomy in Patients with Ulcerative Colitis and Primary Sclerosing Cholangitis. J. Crohns Colitis.

[105] Ishii H., Hata K., Kishikawa J., Anzai H., Otani K., Yasuda K., Nishikawa T., Tanaka T., Tanaka J., Kiyomatsu T. (2016). Incidence of Neoplasias and Effectiveness of Postoperative Surveillance Endoscopy for Patients with Ulcerative Colitis: Comparison of Ileorectal Anastomosis and Ileal Pouch-Anal Anastomosis. World J. Surg. Oncol..

[106] Mark-Christensen A., Erichsen R., Brandsborg S., Rosenberg J., Qvist N., Thorlacius-Ussing O., Hillingsø J., Pachler J.H., Christiansen E.G., Laurberg S. (2018). Long-Term Risk of Cancer Following Ileal Pouch-Anal Anastomosis for Ulcerative Colitis. J. Crohns Colitis.

[107] D’Souza F.R., Lim M., Hainsworth A., Mahadeva U., Ciclitira P.J., Carapeti E. (2011). A Case of Squamous Cell Carcinoma in an Ileoanal Pouch. Colorectal Dis..

[108] Schaffzin D.M., Smith L.E. (2005). Squamous-Cell Carcinoma Developing after an Ileoanal Pouch Procedure: Report of a Case. Dis. Colon Rectum.

[109] Macdonald E., Gee C., Kerr K., Denison A., Keenan R., Binnie N. (2010). Squamous Cell Carcinoma of an Ileo-Anal Pouch. Colorectal Dis..

[110] Pellino G., Kontovounisios C., Tait D., Nicholls J., Tekkis P.P. (2017). Squamous Cell Carcinoma of the Anal Transitional Zone after Ileal Pouch Surgery for Ulcerative Colitis: Systematic Review and Treatment Perspectives. Case Rep. Oncol..

[111] Smart C.J., Gibb A., Radford J. (2012). Burkitt’s Lymphoma of an Ileal Pouch Following Restorative Proctocolectomy. Inflamm. Bowel Dis..

[112] Schwartz L.K., Kim M.K., Coleman M., Lichtiger S., Chadburn A., Scherl E. (2006). Case Report: Lymphoma Arising in an Ileal Pouch Anal Anastomosis after Immunomodulatory Therapy for Inflammatory Bowel Disease. Clin. Gastroenterol. Hepatol..

[113] Jones O.M., McCutcheon J., Herieka E., Fozard J.B. (2007). High-Grade Lymphoma of the Ileoanal Pouch in an HIV-Positive Patient. Colorectal Dis..

[114] Sengul N., Berho M., Baig M.K., Weiss E. (2008). Ileal Pouch Lymphoma Following Restorative Proctocolectomy for Ulcerative Colitis. Inflamm. Bowel Dis..

[115] Frizzi J.D., Rivera D.E., Harris J.A., Hamill R.L. (2000). Lymphoma Arising in an S-Pouch after Total Proctocolectomy for Ulcerative Colitis: Report of a Case. Dis. Colon Rectum.

[116] Nyam D.C., Pemberton J.H., Sandborn W.J., Savcenko M. (1997). Lymphoma of the Pouch after Ileal Pouch-Anal Anastomosis: Report of a Case. Dis. Colon Rectum.

[117] Winter A.M., Koeppen H., Hanauer S.B. (1998). Non-Hodgkin’s Lymphoma Presenting Nine Years after Ileal-Pouch Ileoanal Anastomosis for Ulcerative Colitis. Am. J. Gastroenterol..

[118] Resnick M., Pricolo V., Chen S. (2013). Carcinoid Tumor of the Ileoanal Pouch in a Patient with Ulcerative Colitis. Rhode Isl. Med. J..

[119] Al-Khyatt W., Abercrombie J.F. (2013). Carcinoid Tumour Complicating a Restorative Ileo-Anal Pouch for Ulcerative Colitis. Colorectal Dis..

[120] Lingohr P., Galetin T., Matthaei H., Straub E., Jafari A., Bölke E., Kalff J.C., Vestweber K.-H. (2013). Malignant Melanoma of the Ileo-Anal Pouch Anastomosis after Restorative Proctocolectomy for Ulcerative Colitis: Report of a Case. Eur. J. Med. Res..

[121] Parisian K.R., Lopez R., Shen B. (2013). Chronic Pouch Inflammation and Risk for New-Onset Extraintestinal Cancers in Patients with Restorative Proctocolectomy for Ulcerative Colitis. Inflamm. Bowel Dis..

[122] Heppell J., Weiland L.H., Perrault J., Pemberton J.H., Telander R.L., Beart R.W. (1983). Fate of the Rectal Mucosa after Rectal Mucosectomy and Ileoanal Anastomosis. Dis. Colon Rectum.

[123] O’Connell P.R., Pemberton J.H., Weiland L.H., Beart R.W., Dozois R.R., Wolff B.G., Telander R.L. (1987). Does Rectal Mucosa Regenerate after Ileoanal Anastomosis?. Dis. Colon Rectum.

[124] King D.W., Lubowski D.Z., Cook T.A. (1989). Anal Canal Mucosa in Restorative Proctocolectomy for Ulcerative Colitis. Br. J. Surg..

[125] Gilchrist K.W., Harms B.A., Starling J.R. (1995). Abnormal Rectal Mucosa of the Anal Transitional Zone in Ulcerative Colitis. Arch. Surg..

[126] Sagayama K., Ikeuchi H., Nishigami T., Nakano H., Uchino M., Nakamura M., Noda M., Yanagi H., Yamamura T. (2007). Incidence of and Risk Factors for Dysplasia in Mucosectomy Area in Ulcerative Colitis Patients Undergoing Restorative Proctocolectomy. Int. J. Colorectal Dis..

[127] Fichera A., Ragauskaite L., Silvestri M.T., Elisseou N.M., Rubin M.A., Hurst R.D., Michelassi F. (2007). Preservation of the Anal Transition Zone in Ulcerative Colitis. Long-Term Effects on Defecatory Function. J. Gastrointest. Surg..

[128] Lavery I.C., Sirimarco M.T., Ziv Y., Fazio V.W. (1995). Anal Canal Inflammation after Ileal Pouch-Anal Anastomosis. The Need for Treatment. Dis. Colon Rectum.

[129] Veress B., Reinholt F.P., Lindquist K., Löfberg R., Liljeqvist L. (1995). Long-Term Histomorphological Surveillance of the Pelvic Ileal Pouch: Dysplasia Develops in a Subgroup of Patients. Gastroenterology.

[130] Fruin A.B., El-Zammer O., Stucchi A.F., O’Brien M., Becker J.M. (2003). Colonic Metaplasia in the Ileal Pouch Is Associated with Inflammation and Is Not the Result of Long-Term Adaptation. J. Gastrointest. Surg..

[131] Moskowitz R.L., Shepherd N.A., Nicholls R.J. (1986). An Assessment of Inflammation in the Reservoir after Restorative Proctocolectomy with Ileoanal Ileal Reservoir. Int. J. Colorectal Dis..

[132] Lerch M.M., Braun J., Harder M., Hofstădter F., Schumpelick V., Matern S. (1989). Postoperative Adaptation of the Small Intestine after Total Colectomy and J-Pouch-Anal Anastomosis. Dis. Colon Rectum.

[133] de Silva H.J., Gatter K.C., Millard P.R., Kettlewell M., Mortensen N.J., Jewell D.P. (1990). Crypt Cell Proliferation and HLA-DR Expression in Pelvic Ileal Pouches. J. Clin. Pathol..

[134] Marmorale C., Guercioni G., Siquini W., Freddara U., Santinelli A., Rubini C., Carle F., Landi E. (2003). Evolution of the Changes of the Ileal Pouch Mucosa over a Long Follow-up Period. Hepatogastroenterology.

[135] de Godoy Arashiro R.T., Teixeira M.G., Rawet V., Quintanilha A.G., de Paula H.M., Silva A.Z., Nahas S.C., Cecconello I. (2012). Histopathological Evaluation and Risk Factors Related to the Development of Pouchitis in Patients with Ileal Pouches for Ulcerative Colitis. Clinics.

[136] Garcia-Armengol J., Hinojosa J., Lledo S., Roig J.V., Garcia-Granero E., Martinez B. (1998). Prospective Study of Morphologic and Functional Changes with Time in the Mucosa of the Ileoanal Pouch: Functional Appraisal Using Transmucosal Potential Differences. Dis. Colon Rectum.

[137] Shepherd N.A., Jass J.R., Duval I., Moskowitz R.L., Nicholls R.J., Morson B.C. (1987). Restorative Proctocolectomy with Ileal Reservoir: Pathological and Histochemical Study of Mucosal Biopsy Specimens. J. Clin. Pathol..

[138] Sylvester P.A., Wong N.A.C.S., Myerscough N., Warren B.F., Corfield A.P., Thomas M.G., Durdey P. (2002). Mucin Expression in the Ileoanal Reservoir Reflects Incomplete Mucosal Adaptation. J. Pathol..

[139] Löfberg R., Liljeqvist L., Lindquist K., Veress B., Reinholt F.P., Tribukait B. (1991). Dysplasia and DNA Aneuploidy in a Pelvic Pouch. Report of a Case. Dis. Colon Rectum.

[140] Segal J.P., Oke S., Hold G.L., Clark S.K., Faiz O.D., Hart A.L. (2018). Systematic Review: Ileoanal Pouch Microbiota in Health and Disease. Aliment. Pharmacol. Ther..

[141] Campbell A.P., Merrett M.N., Kettlewell M., Mortensen N.J., Jewell D.P. (1994). Expression of Colonic Antigens by Goblet and Columnar Epithelial Cells in Ileal Pouch Mucosa: Their Association with Inflammatory Change and Faecal Stasis. J. Clin. Pathol..

[142] de Silva H.J., Millard P.R., Soper N., Kettlewell M., Mortensen N., Jewell D.P. (1991). Effects of the Faecal Stream and Stasis on the Ileal Pouch Mucosa. Gut.

[143] Bambury N., Coffey J.C., Burke J., Redmond H.P., Kirwan W.O. (2008). Sulphomucin Expression in Ileal Pouches: Emerging Differences between Ulcerative Colitis and Familial Adenomatous Polyposis Pouches. Dis. Colon Rectum.

[144] Nasmyth D.G., Godwin P.G., Dixon M.F., Williams N.S., Johnston D. (1989). Ileal Ecology after Pouch-Anal Anastomosis or Ileostomy. A Study of Mucosal Morphology, Fecal Bacteriology, Fecal Volatile Fatty Acids, and Their Interrelationship. Gastroenterology.

[145] Duffy M., O’Mahony L., Coffey J.C., Collins J.K., Shanahan F., Redmond H.P., Kirwan W.O. (2002). Sulfate-Reducing Bacteria Colonize Pouches Formed for Ulcerative Colitis but Not for Familial Adenomatous Polyposis. Dis. Colon Rectum.

[146] Kuisma J., Mentula S., Luukkonen P., Jarvinen H., Kahri A., Farkkila M. (2003). Factors Associated with Ileal Mucosal Morphology and Inflammation in Patients with Ileal Pouch-Anal Anastomosis for Ulcerative Colitis. Dis. Colon Rectum.

[147] Apel R., Cohen Z., Andrews C.W., McLeod R., Steinhart H., Odze R.D. (1994). Prospective Evaluation of Early Morphological Changes in Pelvic Ileal Pouches. Gastroenterology.

[148] Herbst F., Ciclitira P.J., Talbot I.C., Nicholls R.J. (2000). Early Changes of Ileoanal Pouch Mucosa in Patients with Ulcerative Colitis. Eur. J. Gastroenterol. Hepatol..

[149] Das P., Smith J.J., Lyons A.P., Tekkis P.P., Clark S.K., Nicholls R.J. (2008). Assessment of the Mucosa of the Indefinitely Diverted Ileo-Anal Pouch. Colorectal Dis..

[150] Smith F.M., Coffey J.C., Kell M.R., O’Sullivan M., Redmond H.P., Kirwan W.O. (2005). A Characterization of Anaerobic Colonization and Associated Mucosal Adaptations in the Undiseased Ileal Pouch. Colorectal Dis..

[151] Gibson G.R., Cummings J.H., Macfarlane G.T. (1991). Growth and Activities of Sulphate-Reducing Bacteria in Gut Contents of Healthy Subjects and Patients with Ulcerative Colitis. FEMS Microbiol. Lett..

[152] Clausen M.R., Tvede M., Mortensen P.B. (1992). Short-Chain Fatty Acids in Pouch Contents from Patients with and without Pouchitis after Ileal Pouch-Anal Anastomosis. Gastroenterology.

[153] Sinha S.R., Haileselassie Y., Nguyen L.P., Tropini C., Wang M., Becker L.S., Sim D., Jarr K., Spear E.T., Singh G. (2020). Dysbiosis-Induced Secondary Bile Acid Deficiency Promotes Intestinal Inflammation. Cell Host Microbe.

[154] Toiyama Y., Araki T., Yoshiyama S., Hiro J., Miki C., Kusunoki M. (2006). The Expression Patterns of Toll-like Receptors in the Ileal Pouch Mucosa of Postoperative Ulcerative Colitis Patients. Surg. Today.

[155] Leal R.F., Coy C.S.R., Ayrizono M.L.S., Fagundes J.J., Milanski M., Saad M.J., Velloso L.A., Góes J.R.N. (2008). Differential Expression of Pro-Inflammatory Cytokines and a pro-Apoptotic Protein in Pelvic Ileal Pouches for Ulcerative Colitis and Familial Adenomatous Polyposis. Tech. Coloproctol..

[156] Leal R.F., Ayrizono M.d.L.S., Milanski M., Fagundes J.J., Moraes J.C., Meirelles L.R., Velloso L.A., Coy C.S.R. (2010). Detection of Epithelial Apoptosis in Pelvic Ileal Pouches for Ulcerative Colitis and Familial Adenomatous Polyposis. J. Transl. Med..

[157] Paiva N.M., Pascoal L.B., Negreiros L.M.V., Portovedo M., Coope A., Ayrizono M. (2018). de L.S.; Coy, C.S.R.; Milanski, M.; Leal, R.F. Ileal Pouch of Ulcerative Colitis and Familial Adenomatous Polyposis Patients Exhibit Modulation of Autophagy Markers. Sci. Rep..

[158] Gullberg K., Lindforss U., Zetterquist H., Stålberg D., Reinholt F.P., Veress B., Tribukait B., Olivecrona H., Löfberg R. (2002). Cancer Risk Assessment in Long-Standing Pouchitis. DNA Aberrations Are Rare in Transformed Neoplastic Pelvic Pouch Mucosa. Int. J. Colorectal Dis..

[159] Coull D.B., Lee F.D., Anderson J.H., McKee R.F., Finlay I.G., Dunlop M.G. (2007). Long-Term Cancer Risk of the Anorectal Cuff Following Restorative Proctocolectomy Assessed by P53 Expression and Cuff Dysplasia. Colorectal Dis..

[160] Elkowitz D., Daum F., Markowitz J., Proccaccino J., Boas E., Cuomo J., Kahn E. (2004). Risk Factors for Carcinoma of the Pelvic Ileal Pouch/Anal Canal in Ulcerative Colitis. Ann. Clin. Lab. Sci..

[161] Jiang W., Shadrach B., Carver P., Goldblum J.R., Shen B., Liu X. (2012). Histomorphologic and Molecular Features of Pouch and Peripouch Adenocarcinoma: A Comparison with Ulcerative Colitis-Associated Adenocarcinoma. Am. J. Surg. Pathol..

[162] Ben-Shachar S., Yanai H., Sherman Horev H., Elad H., Baram L., Issakov O., Tulchinsky H., Pasmanik-Chor M., Shomron N., Dotan I. (2016). MicroRNAs Expression in the Ileal Pouch of Patients with Ulcerative Colitis Is Robustly Up-Regulated and Correlates with Disease Phenotypes. PLoS ONE.

[163] Sherman Horev H., Rabinowitz K.M., Elad H., Barkan R., Ben-Shachar S., Pasmanik Chor M., Dotan I. (2018). Increase in Processing Factors Is Involved in Skewed MicroRNA Expression in Patients with Ulcerative Colitis Who Develop Small Intestine Inflammation after Pouch Surgery. Inflamm. Bowel Dis..

[164] Shen B., Remzi F.H., Lavery I.C., Lashner B.A., Fazio V.W. (2008). A Proposed Classification of Ileal Pouch Disorders and Associated Complications after Restorative Proctocolectomy. Clin. Gastroenterol. Hepatol..

[165] Derikx L.A.A.P., Nissen L.H.C., Oldenburg B., Hoentjen F. (2016). Controversies in Pouch Surveillance for Patients with Inflammatory Bowel Disease. J. Crohns Colitis.

[166] Cairns S.R., Scholefield J.H., Steele R.J., Dunlop M.G., Thomas H.J.W., Evans G.D., Eaden J.A., Rutter M.D., Atkin W.P., Saunders B.P. (2010). Guidelines for Colorectal Cancer Screening and Surveillance in Moderate and High Risk Groups (Update from 2002). Gut.

[167] Annese V., Beaugerie L., Egan L., Biancone L., Bolling C., Brandts C., Dierickx D., Dummer R., Fiorino G., Gornet J.M. (2015). European Evidence-Based Consensus: Inflammatory Bowel Disease and Malignancies. J. Crohn’s Colitis.

[168] Annese V., Daperno M., Rutter M.D., Amiot A., Bossuyt P., East J., Ferrante M., Götz M., Katsanos K.H., Kießlich R. (2013). European Evidence Based Consensus for Endoscopy in Inflammatory Bowel Disease. J. Crohns Colitis.

[169] Shergill A.K., Lightdale J.R., Bruining D.H., Acosta R.D., Chandrasekhara V., Chathadi K.V., Decker G.A., Early D.S., Evans J.A., American Society for Gastrointestinal Endoscopy Standards of Practice Committee (2015). The Role of Endoscopy in Inflammatory Bowel Disease. Gastrointest. Endosc..

[170] Gu J., Remzi F.H., Lian L., Shen B. (2016). Practice Pattern of Ileal Pouch Surveillance in Academic Medical Centers in the United States. Gastroenterol. Rep..

[171] Samaan M.A., Forsyth K., Segal J.P., De Jong D., Vleugels J.L.A., Elkady S., Kabir M., Campbell S., Kok K., Armstrong D.G. (2019). Current Practices in Ileal Pouch Surveillance for Patients With Ulcerative Colitis: A Multinational, Retrospective Cohort Study. J. Crohns Colitis.

[172] Shen B., Kochhar G.S., Kariv R., Liu X., Navaneethan U., Rubin D.T., Cross R.K., Sugita A., D’Hoore A., Schairer J. (2021). Diagnosis and Classification of Ileal Pouch Disorders: Consensus Guidelines from the International Ileal Pouch Consortium. Lancet Gastroenterol. Hepatol..

[173] Schaus B.J., Fazio V.W., Remzi F.H., Bennett A.E., Lashner B.A., Shen B. (2007). Clinical Features of Ileal Pouch Polyps in Patients with Underlying Ulcerative Colitis. Dis. Colon Rectum.

[174] Shen B., Kochhar G.S., Rubin D.T., Kane S.V., Navaneethan U., Bernstein C.N., Cross R.K., Sugita A., Schairer J., Kiran R.P. (2021). Treatment of Pouchitis, Crohn’s Disease, Cuffitis, and Other Inflammatory Disorders of the Pouch: Consensus Guidelines from the International Ileal Pouch Consortium. Lancet Gastroenterol. Hepatol..

[175] Liu Z.-X., Kiran R.P., Bennett A.E., Ni R.-Z., Shen B. (2011). Diagnosis and Management of Dysplasia and Cancer of the Ileal Pouch in Patients with Underlying Inflammatory Bowel Disease. Cancer.

[176] McLaughlin S.D., Clark S.K., Thomas-Gibson S., Tekkis P.P., Ciclitira P.J., Nicholls R.J. (2009). Guide to Endoscopy of the Ileo-Anal Pouch Following Restorative Proctocolectomy with Ileal Pouch-Anal Anastomosis; Indications, Technique, and Management of Common Findings. Inflamm. Bowel Dis..

[177] Shen B. (2016). The Evaluation of Postoperative Patients with Ulcerative Colitis. Gastrointest. Endosc. Clin. N. Am..

[178] Hurlstone D.P., Shorthouse A.J., Cross S.S., Brown S., Sanders D.S., Lobo A.J. (2004). High-Magnification Chromoscopic Pouchoscopy: A Novel in Vivo Technique for Surveillance of the Anal Transition Zone and Columnar Cuff Following Ileal Pouch-Anal Anastomosis. Tech. Coloproctol..

[179] van der Sommen F., de Groof J., Struyvenberg M., van der Putten J., Boers T., Fockens K., Schoon E.J., Curvers W., de With P., Mori Y. (2020). Machine Learning in GI Endoscopy: Practical Guidance in How to Interpret a Novel Field. Gut.

[180] Van Duijvendijk P., Vasen H.F., Bertario L., Bülow S., Kuijpers J.H., Schouten W.R., Guillem J.G., Taat C.W., Slors J.F. (1999). Cumulative Risk of Developing Polyps or Malignancy at the Ileal Pouch-Anal Anastomosis in Patients with Familial Adenomatous Polyposis. J. Gastrointest. Surg..

[181] Hoehner J.C., Metcalf A.M. (1994). Development of Invasive Adenocarcinoma Following Colectomy with Ileoanal Anastomosis for Familial Polyposis Coli. Report of a Case. Dis. Colon Rectum.

[182] Von Herbay A., Stern J., Herfarth C. (1996). Pouch-Anal Cancer after Restorative Proctocolectomy for Familial Adenomatous Polyposis. Am. J. Surg. Pathol..

[183] Malassagne B., Penna C., Parc R. (1995). Adenomatous Polyps in the Anal Transitional Zone after Ileal Pouch-Anal Anastomosis for Familial Adenomatous Polyposis: Treatment by Transanal Mucosectomy and Ileal Pouch Advancement. Br. J. Surg..

[184] Brown S.R., Donati D., Seow-Choen F. (2001). Rectal Cancer after Mucosectomy for Ileoanal Pouch in Familial Adenomatous Polyposis: Report of a Case. Dis. Colon Rectum.

[185] Vuilleumier H., Halkic N., Ksontini R., Gillet M. (2000). Columnar Cuff Cancer after Restorative Proctocolectomy for Familial Adenomatous Polyposis. Gut.

[186] Vrouenraets B.C., Van Duijvendijk P., Bemelman W.A., Offerhaus G.J.A., Slors J.F.M. (2004). Adenocarcinoma in the Anal Canal after Ileal Pouch-Anal Anastomosis for Familial Adenomatous Polyposis Using a Double-Stapled Technique: Report of Two Cases. Dis. Colon Rectum.

[187] Walsh L.G., Kenny B.J., El Bassiouni M., Coffey J.C. (2016). Cancer Arising from the Remnant Mucosa of the Ileoanal Anastomosis Leading to Pouchectomy. BMJ Case Rep..

[188] Ulaş M., Neşşar G., Bostanoğlu A., Aydoğ G., Kayaalp C., Ozoğul Y., Seven C. (2006). Development of Two Cancers in the Same Patient after Ileorectal and Ileal Pouch Anal Anastomosis for Familial Adenomatous Polyposis. Med. Princ. Pract..

[189] Von Roon A.C., Tekkis P.P., Clark S.K., Heriot A.G., Lovegrove R.E., Truvolo S., Nicholls R.J., Phillips R.K.S. (2007). The Impact of Technical Factors on Outcome of Restorative Proctocolectomy for Familial Adenomatous Polyposis. Dis. Colon Rectum.

[190] Wu J.S., McGannon E.A., Church J.M. (1998). Incidence of Neoplastic Polyps in the Ileal Pouch of Patients with Familial Adenomatous Polyposis after Restorative Proctocolectomy. Dis. Colon Rectum.

[191] Ooi B.S., Remzi F.H., Gramlich T., Church J.M., Preen M., Fazio V.W. (2003). Anal Transitional Zone Cancer after Restorative Proctocolectomy and Ileoanal Anastomosis in Familial Adenomatous Polyposis: Report of Two Cases. Dis. Colon Rectum.

[192] Booij K.A.C., Mathus-Vliegen E.M.H., Taminiau J.A.J.M., Ten Kate F.J.W., Slors J.F.M., Tabbers M.M., Aronson D.C. (2010). Evaluation of 28 Years of Surgical Treatment of Children and Young Adults with Familial Adenomatous Polyposis. J. Pediatr. Surg..

[193] Banasiewicz T., Marciniak R., Kaczmarek E., Krokowicz P., Paszkowski J., Lozynska-Nelke A., Gronek P., Plawski A., Drews M. (2011). The Prognosis of Clinical Course and the Analysis of the Frequency of the Inflammation and Dysplasia in the Intestinal J-Pouch at the Patients after Restorative Proctocolectomy Due to FAP. Int. J. Colorectal Dis..

[194] Tonelli F., Ficari F., Bargellini T., Valanzano R. (2012). Ileal Pouch Adenomas and Carcinomas after Restorative Proctocolectomy for Familial Adenomatous Polyposis. Dis. Colon Rectum.

[195] Kennedy R.D., Zarroug A.E., Moir C.R., Mao S.A., El-Youssef M., Potter D.D. (2014). Ileal Pouch Anal Anastomosis in Pediatric Familial Adenomatous Polyposis: A 24-Year Review of Operative Technique and Patient Outcomes. J. Pediatr. Surg..

[196] Lee C.H.A., Kalady M.F., Burke C.A., Mankaney G., Ali Abbass M., Jia X., Church J. (2021). Incidence and Management of Rectal Cuff and Anal Transitional Zone Neoplasia in Patients With Familial Adenomatous Polyposis. Dis. Colon Rectum.

[197] Tytgat G.N., Gopinath N. (1995). Recurrent Polyps in the Ileo-Anal Pouch or Rectum in Familial Adenomatous Polyposis. Eur. J. Cancer.

[198] Nugent K.P., Spigelman A.D., Nicholls R.J., Talbot I.C., Neale K., Phillips R.K.S. (1993). Pouch Adenomas in Patients with Familial Adenomatous Polyposis. Br. J. Surg..

[199] Thompson-Fawcett M.W., Marcus V.A., Redston M., Cohen Z., Mcleod R.S. (2001). Adenomatous Polyps Develop Commonly in the Ileal Pouch of Patients with Familial Adenomatous Polyposis. Dis. Colon Rectum.

[200] Groves C.J., Beveridge I.G., Swain D.J., Saunders B.P., Talbot I.C., Nicholls R.J., Phillips R.K. (2005). Prevalence and Morphology of Pouch and Ileal Adenomas in Familial Adenomatous Polyposis. Dis. Colon Rectum.

[201] Moussata D., Nancey S., Lapalus M.G., Prost B., Chavaillon A., Bernard G., Ponchon T., Saurin J.C. (2008). Frequency and Severity of Ileal Adenomas in Familial Adenomatous Polyposis after Colectomy. Endoscopy.

[202] Tulchinsky H., Keidar A., Strul H., Goldman G., Klausner J.M., Rabau M. (2005). Extracolonic Manifestations of Familial Adenomatous Polyposis after Proctocolectomy. Arch. Surg..

[203] Tajika M., Nakamura T., Nakahara O., Kawai H., Komori K., Hirai T., Kato T., Bhatia V., Baba H., Yamao K. (2009). Prevalence of Adenomas and Carcinomas in the Ileal Pouch after Proctocolectomy in Patients with Familial Adenomatous Polyposis. J. Gastrointest. Surg..

[204] Schulz A.C., Bojarski C., Buhr H.J., Kroesen A.J. (2008). Occurrence of Adenomas in the Pouch and Small Intestine of FAP Patients after Proctocolectomy with Ileoanal Pouch Construction. Int. J. Colorectal Dis..

[205] Goldstein A.L., Kariv R., Klausner J.M., Tulchinsky H. (2015). Patterns of Adenoma Recurrence in Familial Adenomatous Polyposis Patients after Ileal Pouch-Anal Anastomosis. Dig. Surg..

[206] Kariv R., Rosner G., Fliss-Isakov N., Gluck N., Goldstein A., Tulchinsky H., Zelber-Sagi S. (2019). Genotype-Phenotype Associations of APC Mutations With Pouch Adenoma in Patients With Familial Adenomatous Polyposis. J. Clin. Gastroenterol..

[207] Parc Y.R., Olschwang S., Desaint B., Schmitt G., Parc R.G., Tiret E. (2001). Familial Adenomatous Polyposis: Prevalence of Adenomas in the Ileal Pouch after Restorative Proctocolectomy. Ann. Surg..

[208] Ganschow P., Treiber I., Hinz U., Leowardi C., Büchler M.W., Kadmon M. (2015). Residual Rectal Mucosa after Stapled vs. Handsewn Ileal J-Pouch-Anal Anastomosis in Patients with Familial Adenomatous Polyposis Coli (FAP)—A Critical Issue. Langenbecks Arch. Surg..

[209] Makni A., Chebbi F., Rebai W., Ayadi S., Fekih M., Jouini M., Kacem M., Ben Safta Z. (2012). Adenocarcinoma Arising in the “J” Pouch after Total Proctocolectomy for Familial Polyposis Coli. Tunis Med..

[210] Linehan G., Cahill R.A., Kalimuthu S.N., O’Connell F., Redmond H.P., Kirwan W.O. (2008). Adenocarcinoma Arising in the Ileoanal Pouch after Restorative Proctocolectomy for Familial Adenomatous Polyposis. Int. J. Colorectal Dis..

[211] Palkar V.M., deSouza L.J., Jagannath P., Naresh K.N. (1997). Adenocarcinoma Arising in “J” Pouch after Total Proctocolectomy for Familial Polyposis Coli. Indian J. Cancer.

[212] Lee S.H., Ahn B.K., Chang H.-K., Baek S.U. (2009). Adenocarcinoma in Ileal Pouch after Proctocolectomy for Familial Adenomatous Polyposis: Report of a Case. J. Korean Med. Sci..

[213] Mackni A., Lefevre J.H., Ewald J., Chafai N., Tiret E., Parc Y. (2011). Revision of an Ileoanal Pouch for Recurrent Pouch Adenomas in a Patient with Familial Adenomatous Polyposis: A Case Report. Colorectal Dis..

[214] Bassuini M.M., Billings P.J. (1996). Carcinoma in an Ileoanal Pouch after Restorative Proctocolectomy for Familial Adenomatous Polyposis. Br. J. Surg..

[215] Tajika M., Nakamura T., Bhatia V., Komori K., Kato T., Yamao K. (2009). Ileal Pouch Adenocarcinoma after Proctocolectomy for Familial Adenomatous Polyposis. Int. J. Colorectal Dis..

[216] Cherki S., Glehen O., Moutardier V., François Y., Gilly F.N., Vignal J. (2003). Pouch Adenocarcinoma after Restorative Proctocolectomy for Familial Adenomatous Polyposis. Colorectal Dis..

[217] Campos F.G., Habr-Gama A., Kiss D.R., da Silva E.V., Rawet V., Imperiale A.R., Perez R., da Silva J.H., Sousa A.H.S., Gama-Rodrigues J. (2005). Adenocarcinoma after Ileoanal Anastomosis for Familial Adenomatous Polyposis: Review of Risk Factors and Current Surveillance apropos of a Case. J. Gastrointest. Surg..

[218] Lovegrove R.E., Constantinides V.A., Heriot A.G., Athanasiou T., Darzi A., Remzi F.H., Nicholls R.J., Fazio V.W., Tekkis P.P. (2006). A Comparison of Hand-Sewn versus Stapled Ileal Pouch Anal Anastomosis (IPAA) Following Proctocolectomy: A Meta-Analysis of 4183 Patients. Ann. Surg..

[219] Friederich P., de Jong A.E., Mathus-Vliegen L.M., Dekker E., Krieken H.H., Dees J., Nagengast F.M., Vasen H.F.A. (2008). Risk of Developing Adenomas and Carcinomas in the Ileal Pouch in Patients with Familial Adenomatous Polyposis. Clin. Gastroenterol. Hepatol..

[220] Campos F.G., Imperiale A.R., Seid V.E., Perez R.O., da Silva e Sousa A.H., Kiss D.R., Habr-Gama A., Cecconello I. (2009). Rectal and Pouch Recurrences after Surgical Treatment for Familial Adenomatous Polyposis. J. Gastrointest. Surg..

[221] Boostrom S.Y., Mathis K.L., Pendlimari R., Cima R.R., Larson D.W., Dozois E.J. (2013). Risk of Neoplastic Change in Ileal Pouches in Familial Adenomatous Polyposis. J. Gastrointest. Surg..

[222] Pommaret E., Vienne A., Lefevre J.H., Sogni P., Florent C., Desaint B., Parc Y. (2013). Prevalence and Risk Factors for Adenomas in the Ileal Pouch and the Afferent Loop after Restorative Proctocolectomy for Patients with Familial Adenomatous Polyposis. Surg. Endosc..

[223] Slors J.F., Ponson A.E., Taat C.W., Bosma A. (1995). Risk of Residual Rectal Mucosa after Proctocolectomy and Ileal Pouch-Anal Reconstruction with the Double-Stapling Technique. Postoperative Endoscopic Follow-up Study. Dis. Colon Rectum.

[224] Iida M., Itoh H., Matsui T., Mibu R., Iwashita A., Fujishima M. (1989). Ileal Adenomas in Postcolectomy Patients with Familial Adenomatosis Coli/Gardner’s Syndrome. Incidence and Endoscopic Appearance. Dis. Colon Rectum.

[225] Wolfstein I.H., Bat L., Neumann G. (1982). Regeneration of Rectal Mucosa and Recurrent Polyposis Coli after Total Colectomy and Ileoanal Anastomosis. Arch. Surg..

[226] Will O.C.C., Deheragoda M., Phillips R.K.S., Clark S.K., Tomlinson I.P.M. (2011). The Role of Cell Proliferation and Crypt Fission in Adenoma Aggressiveness: A Comparison of Ileoanal Pouch and Rectal Adenomas in Familial Adenomatous Polyposis. Colorectal Dis..

[227] Church J. (2005). Ileoanal Pouch Neoplasia in Familial Adenomatous Polyposis: An Underestimated Threat. Dis. Colon Rectum.

[228] Natori H., Utsunomiya J., Yamamura T., Benno Y., Uchida K. (1992). Fecal and Stomal Bile Acid Composition after Ileostomy or Ileoanal Anastomosis in Patients with Chronic Ulcerative Colitis and Adenomatosis Coli. Gastroenterology.

[229] De Silva H.J., Millard P.R., Kettlewell M., Mortensen N.J., Prince C., Jewell D.P. (1991). Mucosal Characteristics of Pelvic Ileal Pouches. Gut.

[230] Nasmyth D.G., Johnston D., Williams N.S., King R.F., Burkinshaw L., Brooks K. (1989). Changes in the Absorption of Bile Acids after Total Colectomy in Patients with an Ileostomy or Pouch-Anal Anastomosis. Dis. Colon Rectum.

[231] Johnson J.A., Talton D.S., Poole G.V. (1993). Adenocarcinoma of a Brooke Ileostomy for Adenomatous Polyposis Coli. Am. J. Gastroenterol..

[232] Metzger P.P., Slappy A.L.J., Chua H.K., Menke D.M. (2008). Adenocarcinoma Developing at an Ileostomy: Report of a Case and Review of the Literature. Dis. Colon Rectum.

[233] Friederich P., Berkhout M., Roelofs H.M.J., van Goor H., van Krieken J.H.J.M., Peters W.H.M., Nagengast F.M. (2006). Decreased Levels of Mucosal Detoxification Enzymes in the Pouch of Patients with Familial Adenomatous Polyposis. Br. J. Surg..

[234] Friederich P., van Heumen B.W.H., Nagtegaal I.D., Berkhout M., van Krieken J.H.J.M., Peters W.H.M., Nagengast F.M. (2007). Increased Epithelial Cell Proliferation in the Ileal Pouch Mucosa of Patients with Familial Adenomatous Polyposis. Virchows Arch..

[235] Will O.C.C., Robinson J., Günther T., Phillips R.K.S., Clark S.K., Tomlinson I. (2008). APC Mutation Spectrum in Ileoanal Pouch Polyps Resembles That of Colorectal Polyps. Br. J. Surg..

[236] van Leerdam M.E., Roos V.H., van Hooft J.E., Dekker E., Jover R., Kaminski M.F., Latchford A., Neumann H., Pellisé M., Saurin J.-C. (2019). Endoscopic Management of Polyposis Syndromes: European Society of Gastrointestinal Endoscopy (ESGE) Guideline. Endoscopy.

[237] Patel N.J., Ponugoti P.L., Rex D.K. (2016). Cold Snare Polypectomy Effectively Reduces Polyp Burden in Familial Adenomatous Polyposis. Endosc. Int. Open.

[238] Tajika M., Niwa Y., Bhatia V., Tanaka T., Ishihara M., Yamao K. (2013). Risk of Ileal Pouch Neoplasms in Patients with Familial Adenomatous Polyposis. World J. Gastroenterol..

[239] Saurin J.-C., Napoleon B., Gay G., Ponchon T., Arpurt J.-P., Boustiere C., Boyer J., Canard J.-M., Dalbies P.-A., Escourrou J. (2005). Endoscopic Management of Patients with Familial Adenomatous Polyposis (FAP) Following a Colectomy. Endoscopy.

[240] Hurlstone D.P., Saunders B.P., Church J.M. (2008). Endoscopic Surveillance of the Ileoanal Pouch Following Restorative Proctocolectomy for Familial Adenomatous Polyposis. Endoscopy.

[241] Man R., Fraser C., Saunders B.P. (2006). Retroflexion in Flexible Pouchoscopy Can Increase Adenoma Detection in Patients with Familial Adenomatous Polyposis after Restorative Proctocolectomy. Gut.

[242] Douma K.F.L., Bleiker E.M.A., Aaronson N.K., Cats A., Gerritsma M.A., Gundy C.M., Vasen H.F.A. (2010). Long-Term Compliance with Endoscopic Surveillance for Familial Adenomatous Polyposis. Colorectal Dis..

[243] Duff S.E., O’Dwyer S.T., Hultén L., Willén R., Haboubi N.Y. (2002). Dysplasia in the Ileoanal Pouch. Colorectal Dis..

[244] Zahid A., Kumar S., Koorey D., Young C.J. (2015). Pouch Adenomas in Familial Adenomatous Polyposis after Restorative Proctocolectomy. Int. J. Surg..

[245] Riddell R.H., Goldman H., Ransohoff D.F., Appelman H.D., Fenoglio C.M., Haggitt R.C., Ahren C., Correa P., Hamilton S.R., Morson B.C. (1983). Dysplasia in Inflammatory Bowel Disease: Standardized Classification with Provisional Clinical Applications. Hum. Pathol..

[246] Liu Z.-X., Liu X.-L., Patil D.T., Lian L., Kiran R.P., Remzi F.H., Ni R.-Z., Shen B. (2012). Clinical Significance of Indefinite for Dysplasia on Pouch Biopsy in Patients with Underlying Inflammatory Bowel Disease. J. Gastrointest. Surg..

[247] Royston D.J., Warren B.F. (2011). Are We Reporting Ileal Pouch Biopsies Correctly?. Colorectal Dis..

[248] Van der Ploeg V.A., Maeda Y., Faiz O.D., Hart A.L., Clark S.K. (2018). Standardising Assessment and Documentation of Pouchoscopy. Frontline Gastroenterol..

[249] Coull D.B., Lee F.D., Henderson A.P., Anderson J.H., McKee R.F., Finlay I.G. (2003). Risk of Dysplasia in the Columnar Cuff after Stapled Restorative Proctocolectomy. Br. J. Surg..

[250] Saigusa N., Choi H.-J., Wexner S.D., Woodhouse S.L., Singh J.J., Weiss E.G., Nogueras J.J., Belin B. (2003). Double Stapled Ileal Pouch Anal Anastomosis (DS-IPAA) for Mucosal Ulcerative Colitis (MUC): Is There a Correlation between the Tissue Type in the Circular Stapler Donuts and in Follow-up Biopsy?. Colorectal Dis..

